# Optimized CRISPR/Cas9-mediated *in vivo* genome engineering applicable to monitoring dynamics of endogenous proteins in the mouse neural tissues

**DOI:** 10.1038/s41598-019-47721-4

**Published:** 2019-08-05

**Authors:** Takahiko Matsuda, Izumi Oinuma

**Affiliations:** 1 Laboratory of Cell and Molecular Biology, Graduate School of Life Science, University of Hyogo, 3-2-1 Kouto, Kamigori-Cho, Ako-Gun, Hyogo 678-1297 Japan; 2 Institute for Integrated Cell-Material Sciences (iCeMS), Kyoto University, Yoshida-Honmachi, Sakyo-Ku, Kyoto 606-8501 Japan

**Keywords:** Genetic engineering, Neuronal development

## Abstract

To analyze the expression, localization, and functional dynamics of target proteins *in situ*, especially in living cells, it is important to develop a convenient, versatile, and efficient method to precisely introduce exogenous genes into the genome, which is applicable for labeling and engineering of the endogenous proteins of interest. By combining the CRISPR/Cas9 genome editing technology with an electroporation technique, we succeeded in creating knock-in alleles, from which GFP (RFP)-tagged endogenous proteins are produced, in neurons and glial cells *in vivo* in the developing mouse retina and brain. Correct gene targeting was confirmed by single-cell genotyping and Western blot analysis. Several gene loci were successfully targeted with high efficiency. Moreover, we succeeded in engineering the mouse genome to express foreign genes from the endogenous gene loci using a self-cleaving 2A peptide. Our method could be used to monitor the physiological changes in localization of endogenous proteins and expression levels of both mRNA and protein at a single cell resolution. This work discloses a powerful and widely applicable approach for visualization and manipulation of endogenous proteins in neural tissues.

## Introduction

In order to analyze the expression, localization and functional dynamics of target proteins *in situ*, it is essential to selectively label target proteins of interest. Several methods are widely used to label target proteins. These methods include the use of antibodies specific to target proteins and the transfection of cDNA encoding “tagged” recombinant proteins into cells. The former cannot be used for live cells and tissues, except for the proteins that are localized on the cell surface. Moreover, antibodies specific to target proteins of interest may not always be available. It is not easy to faithfully reproduce the expression levels and patterns of target proteins by transfecting exogenous genes using the latter method. Moreover, it is possible that overexpression of exogenous genes causes undesirable side effects. For example, in the case of rhodopsin, a light-sensitive G-protein coupled receptor in rod photoreceptors, overexpression of exogenous *rhodopsin* in transgenic mice alters the structure and function of rod photoreceptors, and results in retinal degeneration^1–4^.

Inserting a cDNA encoding a tagged recombinant protein into a targeted gene locus via homologous recombination in mouse embryonic stem (ES) cells and subsequently generating transgenic mice is an ideal approach to labeling endogenous proteins^5^. This method could be utilized to overcome the issues stated above. However, gene knock-in experiments in mouse ES cells are usually costly, labor-intensive, and time-consuming. In addition, this technique was only applicable to a limited number of cell lines including mouse ES cells, in which homologous recombination is observed with the high frequency of 1/10 to 1/10^3^. Even in human ES and induced pluripotent stem (iPS) cells, gene knock-in is quite difficult because of low efficiency of spontaneous homologous recombination^6^. Typically, homologous recombination only occurs in cells at a frequency of ~1/10^6^.

Over the past several years, the situation has radically changed with the development of programmable endonucleases, including zinc finger nucleases^7–9^, TALEN^10–12^, and CRISPR/Cas9^13–15^, that specifically cleave the genome at one site. It has been shown that the efficiency of homologous recombination dramatically increases upon the targeted genome cleavage by the programmable nucleases^16^. The genome editing technology using the programmable nucleases has been applied to a variety of cell lines. There have been many reports on the convenient creation of genetically modified animals by injecting the programmable nucleases into the fertilized eggs of various animal species including mice, rats, and zebrafish^17^. More recently, CRISPR/Cas9 genome editing technology was used to introduce non-homologous end joining (NHEJ)-mediated indel mutations into genomes in neurons *in vitro* and *in vivo*^18–22^.

In this study, by combining the CRISPR/Cas9-mediated genome editing technology with an electroporation technique, we tried to label endogenous proteins of interest with fluorescent proteins *in vivo* in the mouse retina and brain by inserting the reporter-tagged cDNAs into the target gene loci. Unlike cultured cell lines which can proliferate infinitely, or fertilized eggs which give rise to adult bodies after many cell divisions, neurons *in vivo* do not clonally expand. Therefore, it is almost impossible to use the correctly targeted neurons for further studies after genotyping. To overcome this problem, we planned to develop a method to identify the correctly targeted neurons without genotyping. We focused on the fact that there is often no promoter activity around the exons encoding the C-termini of proteins. We expected that the knock-in targeting vectors, which correspond to the genomic DNAs encoding the C-termini of proteins, do not express fluorescent reporters by themselves, but that the reporter genes are turned on when the targeting vectors are correctly integrated into the genome by CRISPR/Cas9-mediated homologous recombination.

Here we show that the efficiency of CRISPR/Cas9-mediated gene knock-in in the developing mouse retina and brain is quite high, reaching up to ~10% of the transfected cells at some gene loci, and that the “C-terminal fusion approach” worked well with few false-positive signals.

## Results

### Tagging endogenous rhodopsin with GFP in rod photoreceptors in the mouse retina by *in vivo* gene targeting

**Figure 1:**
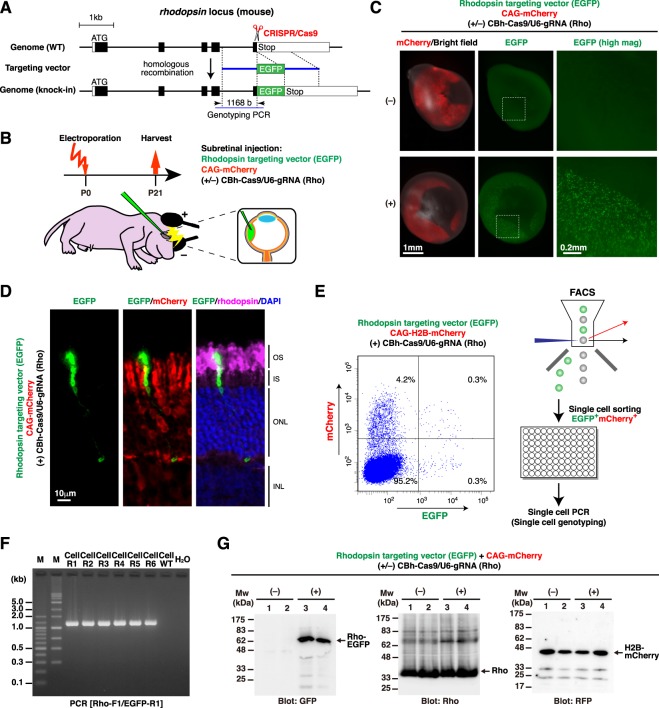
Tagging endogenous rhodopsin with EGFP in the postnatal mouse retina by CRISPR/Cas9-mediated *in vivo* gene targeting. (**A**) Structures of the mouse *rhodopsin* locus and the knock-in targeting vector to produce a rhodopsin-EGFP fusion protein. The predicted Cas9-gRNA cutting position is indicated with a scissor symbol. (**B**) Experimental design for *in vivo* electroporation to the developing mouse retina. (**C**) Mouse retinas were co-electroporated at P0 with three plasmids: Rhodopsin targeting vector (EGFP), CAG-mCherry, and CBh-Cas9 with (+) or without (−) gRNA for *rhodopsin*. Fluorescent images of intact retinas harvested at P21 are shown. White dashed rectangles indicate the regions that are presented at higher magnification in the rightmost panels. (**D**) Retinas shown in (**C**) were sectioned and stained with anti-GFP (green), anti-RFP (red), and anti-rhodopsin (magenta) antibodies. Cell nuclei were visualized with DAPI (blue). (**E**) Isolation of EGFP-positive cells by FACS for single-cell genotyping. P0 mouse retinas were electroporated with Rhodopsin targeting vector (EGFP), CAG-H2B-mCherry, and CBh-Cas9/U6-gRNA (Rho). The retinas were harvested at P21, dissociated into single cells by enzymatic digestion, and EGFP/H2B-mCherry-double positive cells were purified by FACS. EGFP-positive/H2B-mCherry-negative population appeared to represent the cell debris (i.e. detached outer segments). (**F**) Collected single cells (Cell R1-R6) in (**E**) were subjected to genomic PCR with the PCR primers shown in (**A**). An EGFP-negative cell (Cell WT) was used as a negative control. (**G**) Western blot analysis of retinas electroporated with the indicated plasmids at P0 and harvested at P21. Four independently electroporated retinas were analyzed (1 retina per lane), and membranes were probed with anti-GFP, anti-rhodopsin, and anti-RFP antibodies. A rhodopsin-EGFP fusion protein (~67 kDa) was detected only in the presence of CBh-Cas9/U6-gRNA (Rho) (lanes 3, 4). OS, outer segment; IS, inner segment; ONL, outer nuclear layer; INL, inner nuclear layer.

To determine whether and to what extent CRISPR/Cas9-mediated gene knock-in could occur *in vivo* in the postnatal mouse retina, we first chose the *rhodopsin* locus as a target. Rhodopsin is abundantly and selectively expressed in rod photoreceptors in the retina, and within rod photoreceptors, it is predominantly localized in the outer segments^23^. The CRISPR guide RNA (gRNA) was designed to target the genome sequence just after the stop codon in the last exon of the *rhodopsin* gene. The homologous recombination targeting vector (Rhodopsin targeting vector (EGFP)) had ~1 kb homology arms at the 5′ and 3′ ends of the EGFP cDNA to produce a rhodopsin-EGFP fusion protein upon insertion into the *rhodopsin* locus (Fig. 1A, Supplementary Fig. S1A). We designed the targeting vector not to contain the promoter activity so that it could not express EGFP by itself. Once the targeting vector is correctly inserted into the target locus with the aid of CRISPR/Cas9, a fusion protein between rhodopsin and EGFP is produced from the endogenous *rhodopsin* locus.

Postnatal day 0 (P0) mouse retinas were co-electroporated *in vivo* with Rhodopsin targeting vector (EGFP), CAG-mCherry, and the CRISPR construct (CBh-Cas9/U6-gRNA (Rho)) (Fig. 1B). When the transfected retinas were harvested at P21, many bright EGFP-positive cells were observed in intact retinas only in the presence of the CRISPR construct (Fig. 1C). EGFP signals were exclusively detected in rod photoreceptors in the outer nuclear layer (ONL) in retinal sections and were localized predominantly in the rod outer segments and to a lesser extent in the rod inner segments, with little or no expression in rod cell bodies (Fig. 1D, Supplementary Fig. S1B). There were no mCherry-positive cells that expressed EGFP in the inner nuclear layer (INL) and the ganglion cell layer (GCL). This expression pattern of EGFP was consistent with that of endogenous rhodopsin, suggesting that endogenous rhodopsin was correctly tagged with EGFP in the postnatal mouse retina by *in vivo* gene targeting. The number of EGFP-positive cells among the transfected cells (mCherry-positive cells) was estimated to be ~10% on average.

Correct integration of the targeting vector into the *rhodopsin* locus was verified by single-cell genotyping and Western blot analysis. For single-cell genotyping, electroporated retinas were dissociated into single cells by enzymatic digestion and subjected to fluorescence-activated cell sorting (FACS) to collect EGFP/mCherry double-positive cells in 96 well plates (1 cell per well) (Fig. 1E). Polymerase chain reaction (PCR) analysis of the sorted cells revealed that most of the EGFP-positive cells underwent correct gene targeting (Fig. 1F). Western blot analysis of harvested intact retinas also showed that the *rhodopsin* locus was correctly modified to produce a fusion protein (~67 kDa) composed of rhodopsin (~39 kDa) and EGFP (~28 kDa) only in the presence of the CRISPR construct (Fig. 1G).

The knock-in efficiency at the *rhodopsin* locus was dependent on the length of homology arms of the targeting vector. The highest knock-in efficiency was observed when both 5′ and 3′ homology arms of the targeting vector are ~1 kb (Supplementary Fig. S2).

### Biallelic gene knock-in at the *rhodopsin* locus

**Figure 2:**
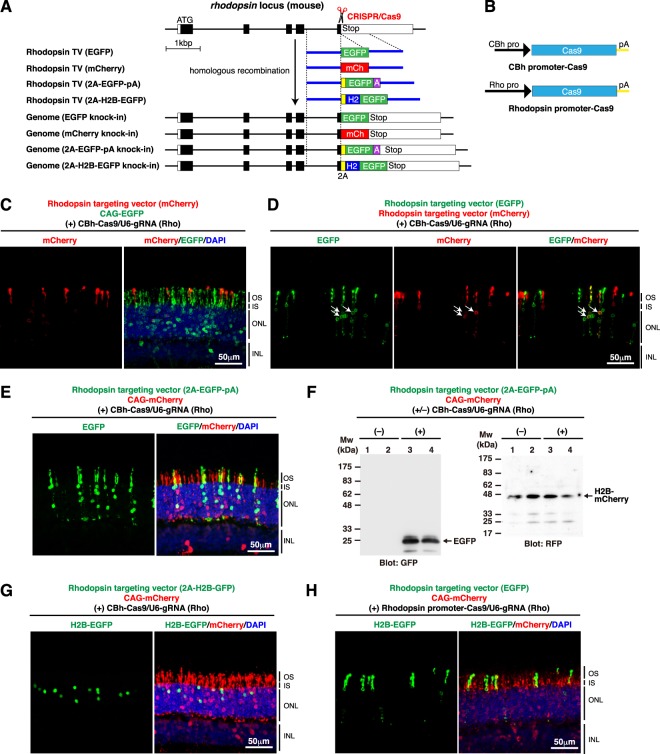
Insertion of various fluorescent protein reporters into the *rhodopsin* locus *in vivo* in the mouse retina. (**A**) Structures of the knock-in targeting vectors to generate *rhodopsin-EGFP*, *rhodopsin-mCherry*, *rhodopsin-2A-EGFP*, and *rhodopsin-2A-H2B-EGFP* fusion genes, respectively. (**B**) Cas9 expression vectors carrying the ubiquitous CBh promoter and the photoreceptor-specific rhodopsin promoter, respectively. (**C**) Tagging endogenous rhodopsin with mCherry in the retina. Mouse retinas were co-electroporated with the indicated plasmids at P0 and harvested at P21. Retinal sections were stained with anti-GFP (green) and anti-RFP (red) antibodies, and DAPI (blue). (**D**) Biallelic gene knock-in at the *rhodopsin* locus in the retina. Mouse retinas were co-electroporated with the indicated plasmids at P0 and harvested at P21. Retinal sections were stained with anti-GFP (green) and anti-RFP (red) antibodies. White arrows indicate EGFP/mCherry double-positive photoreceptors. (**E**) Production of a free EGFP protein from the endogenous *rhodopsin* locus using the 2 A sequence. Mouse retinas were co-electroporated with the indicated plasmids at P0 and harvested at P21. Retinal sections were stained with anti-GFP (green) and anti-RFP (red) antibodies and DAPI (blue). (**F**) Western blot analysis of electroporated retinas shown in (**E**). Four independently transfected retinas were analyzed (1 retina per lane) and membranes were probed with anti-GFP and anti-RFP antibodies. Production of a free EGFP protein (~28 kDa) was observed (lanes 3, 4). (**G**) Expression of a free H2B-EGFP protein from the endogenous *rhodopsin* locus using the 2 A sequence. Mouse retinas were co-electroporated with the indicated plasmids at P0 and harvested at P21. Retinal sections were stained with anti-GFP (green) and anti-RFP (red) antibodies and DAPI (blue). (**H**) Gene knock-in using Cas9 expressed from the rhodopsin promoter. Mouse retinas were co-electroporated with the indicated plasmids at P0 and harvested at P21. Retinal sections were stained with anti-GFP (green) and anti-RFP (red) antibodies and DAPI (blue). OS, outer segment; IS, inner segment; ONL, outer nuclear layer; INL, inner nuclear layer.

To determine whether both alleles of *rhodopsin* can be modified by *in vivo* gene targeting, another targeting vector, which was designed to produce a rhodopsin-mCherry fusion protein from the endogenous *rhodopsin* locus, was constructed (Fig. 2A). Rhodopsin-targeting vector (mCherry) exhibited comparable knock-in efficiency to that of Rhodopsin targeting vector (EGFP) (Fig. 2C). When these two targeting vectors were co-electroporated together with the CRISPR construct into P0 mouse retinas, about 10% of the rod photoreceptors that expressed the fluorescent reporters were double-positive for EGFP and mCherry, indicating that biallelic gene knock-in occurs at the *rhodopsin* locus (Fig. 2D).

### Engineering the *rhodopsin* locus

Taking advantage of the high efficiency of CRISPR/Cas9-mediated *in vivo* gene targeting at the *rhodopsin* locus in the mouse retina, we tried to engineer the mouse genome to express foreign genes from the *rhodopsin* locus. For this purpose, a self-cleaving 2A peptide was used to express multiple proteins from a single locus (Fig. 2A). When the targeting vector encoding 2A-EGFP was used with the CRISPR construct, EGFP expression was observed exclusively in rod photoreceptors, but the fluorescent signals were barely seen in the outer segments and localized mainly in the inner segments, cell bodies, and synapse regions (Fig. 2E). Western blot analysis confirmed the production of a free EGFP protein (~28 kDa), but not a rhodopsin-EGFP fusion protein (~67 kDa), in the retina (Fig. 2F). On the other hand, when the targeting vector encoding 2A-H2B-EGFP, a fusion between histone H2B and EGFP (Fig. 2A), was used, EGFP signals were detected only in rod cell nuclei (Fig. 2G). The data show that *in vivo* gene targeting is a powerful approach to stably express exogenous genes of interest from endogenous gene loci.

### Cell type-specific gene knock-in in the retina

The frequency of *in vivo* gene targeting could be affected by various factors, such as the cell-cycle state of the transfected cells. Using the *rhodopsin* locus as a model, we tried to determine the optimal timing of CRISPR/Cas9-mediated genome cleavage for gene knock-in during mouse retinal development. To control the timing of the expression of Cas9 nuclease in the retina, the rhodopsin promoter, instead of the ubiquitous CBh promoter^24,25^, was used (Fig. 2B). The rhodopsin promoter is specific to postmitotic rod photoreceptors and is inactive in proliferating retinal progenitors^26,27^ (Supplementary Fig. S3). Plasmid DNAs electroporated to P0 mouse retina are normally introduced into undifferentiated retinal progenitors, which give rise to neurons (rod photoreceptors, bipolar cells, and amacrine cells) and Müller glial cells after a few rounds of cell division^28^. Therefore, the time course of genome cleavage mediated by Rhodopsin promoter-Cas9 should be entirely different from that by CBh-Cas9. Unexpectedly, the efficiency of EGFP knock-in at the *rhodopsin* locus mediated by Rhodopsin promoter-Cas9 was almost comparable to that mediated by CBh-Cas9 (Fig. 2H). The data suggest that gene knock-in could occur in postmitotic neurons in the retina and that cell division is not necessarily required for homologous recombination. The data also indicates that “cell type-specific gene knock-in” is possible when cell type-specific promoters are used to express Cas9.

### Tagging endogenous glutamine synthetase in Müller glia in the mouse retina

**Figure 3:**
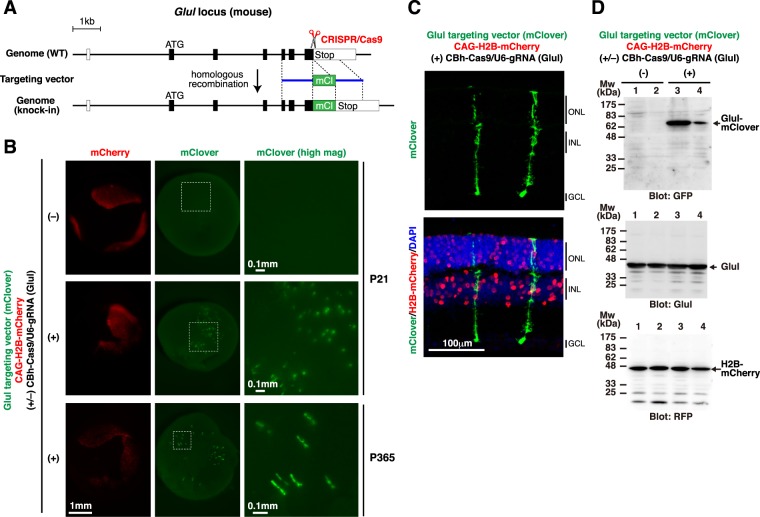
Tagging endogenous glutamine synthetase with mClover in the postnatal mouse retina. (**A**) Structures of the mouse *Glul* locus and the knock-in targeting vector to produce a Glul-mClover fusion protein. The predicted Cas9-gRNA cutting position is indicated with a scissor symbol. (**B**) Mouse retinas were co-electroporated at P0 with Glul targeting vector (mClover), CAG-H2B-mCherry, and CBh-Cas9 with (+) or without (-) gRNA for *Glul*. Fluorescent images of intact retinas harvested at P21 and P365 are shown. White dashed rectangles indicate the regions that are presented at higher magnification in the rightmost panels. (**C**) Retinas shown in (**B**) were sectioned, and stained with anti-GFP (green) and anti-RFP (red) antibodies and DAPI (blue). (**D**) Western blot analysis of retinas electroporated at P0 and harvested at P21. Four independently transfected retinas were analyzed (1 retina per lane), and membranes were probed with anti-GFP, anti-Glul, and anti-RFP antibodies. A Glul-mClover fusion protein (~71 kDa) was detected only in the presence of CBh-Cas9/U6-gRNA (Glul) (lanes 3, 4). ONL, outer nuclear layer; INL, inner nuclear layer; GCL, ganglion cell layer.

To determine if the *in vivo* gene targeting technique could be applied to other gene loci and other cell types in the retina, we chose the *glutamine synthetase* (*Glul*) locus as a target. *Glul* is expressed exclusively in Müller glia in the retina^29^. The CRISPR gRNA was designed to target the genome sequence around the stop codon of the *Glul* gene, and the targeting vector was designed to express a Glul-mClover fusion protein upon insertion into the *Glul* locus (Fig. 3A, Supplementary Fig. S4). This targeting vector also does not contain the promoter region and thereby cannot express the reporter by itself. In the mouse retina that was co-electroporated with Glul targeting vector (mClover), CAG-H2B-mCherry, and the CRISPR construct (CBh-Cas9/U6-gRNA(Glul)), tens of mClover-positive cells were observed at P21 in a CRISPR construct-dependent manner (Fig. 3B). Fluorescent signals were detected even in one-year-old mouse retinas (at P365). Retinal sections showed that mClover expression was detected only in Müller glia (Fig. 3C). Single-cell genotyping and Western blot analysis confirmed the correct gene knock-in and the production of a Glul-mClover fusion protein in the retina (Fig. 3D, Supplementary Fig. S4). The number of mClover-positive cells among the transfected cells (mCherry-positive cells) was ~2%, as determined by FACS. Considering that approximately 10% of the retinal cells transfected at P0 become Müller glia^28^, the knock-in efficiency was estimated to be ~20%.

### Monitoring light-dependent translocation of endogenous arrestin in the mouse retina

**Figure 4:**
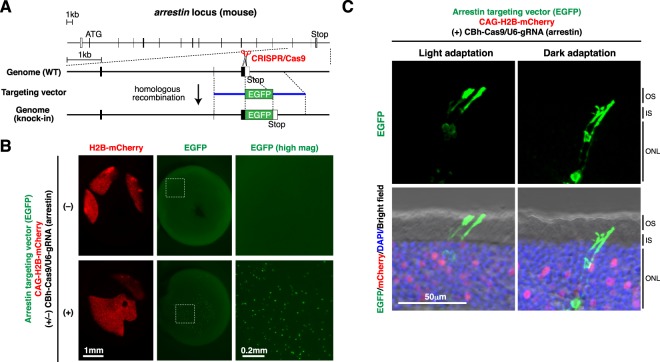
Tagging endogenous arrestin with EGFP in the postnatal mouse retina. (**A**) Structures of the mouse *arrestin* locus and the knock-in targeting vector to produce an arrestin-EGFP fusion protein. The predicted Cas9-gRNA cutting position is indicated with a scissor symbol. (**B**) Mouse retinas were co-electroporated at P0 with Arrestin targeting vector (EGFP), CAG-H2B-mCherry, and CBh-Cas9 with (+) or without (−) gRNA for *arrestin*. Fluorescent images of intact retinas harvested at P21 are shown. CRISPR/Cas9-dependent gene knock-in at the *arrestin* locus occurred at a frequency of ~1% in the electroporated retinas. White dashed rectangles indicate the regions that are presented at higher magnification in the rightmost panels. (**C**) Retinas were collected from the light-adapted (left panels) or dark-adapted mice (right panels), sectioned, and stained with anti-GFP (green) and anti-RFP (red) antibodies and DAPI (blue). EGFP signals were detected only in rod photoreceptors. In the light-adapted retinas, EGFP signals were predominantly localized in the outer segments of rod photoreceptors, while in the dark-adapted retinas, EGFP signals were localized in the inner segments and cell bodies but not in the outer segments of rod photoreceptors. OS, outer segment; IS, inner segment; ONL, outer nuclear layer.

One of the advantages of *in vivo* gene targeting is that it can be used to label endogenous proteins of interest with fluorescent reporters to monitor their dynamic movement within cells. To demonstrate this, we chose arrestin as a target. Arrestin is exclusively expressed in photoreceptors in the retina, and its localization within photoreceptors dynamically changes in response to light illumination. The CRISPR gRNA was designed to target the genome sequence around the stop codon of the *arrestin* gene, and the targeting vector was designed to express an arrestin-EGFP C-terminal fusion protein from the endogenous locus (Fig. 4A, Supplementary Fig. S5). CRISPR/Cas9-dependent gene knock-in at the *arrestin* locus occurred at a frequency of ~1% in the electroporated retinas, and EGFP signals were detected only in rod photoreceptors (Fig. 4B,C). Importantly, in the light-adapted retinas, EGFP signals were predominantly localized in the outer segments of rod photoreceptors, while in the dark-adapted retinas, EGFP signals were localized in the inner segments and cell bodies but not in the outer segments of rod photoreceptors (Fig. 4C). The light-dependent dynamic localization patterns of EGFP within rod photoreceptors were consistent well with those of endogenous arrestin^30^.

### Tagging endogenous synaptic proteins in the mouse retina

Another advantage of *in vivo* gene targeting is that without adverse effects on cells, it can be used to label the endogenous proteins whose overexpression influences cellular functions. Synapses in live neurons are often visualized by transfecting cDNAs encoding fluorescent protein-tagged pre- and post-synaptic proteins, synaptophysin (also called Syp) and PSD-95, respectively. In many cases, however, exogenous expression of tagged Syp and PSD-95 results in “overexpression” and morphology and function of transfected neurons are significantly affected^31,32^. Therefore, to study their dynamic behavior under “physiological” conditions in neurons, it is important to label endogenous Syp and PSD-95 with fluorescent reporters.

**Figure 5:**
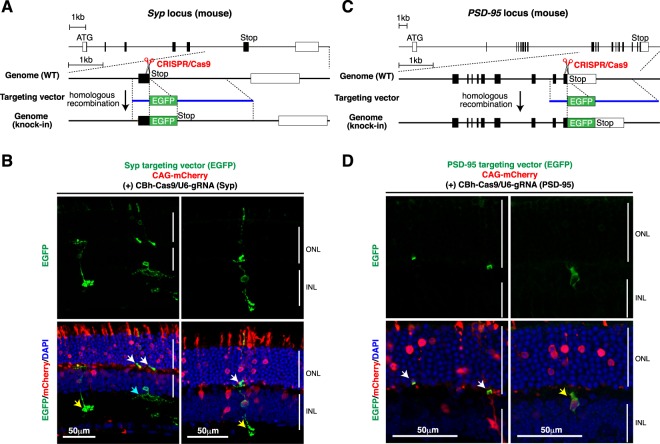
Tagging endogenous synaptic proteins in the mouse retina. (**A**,**B**) Tagging endogenous synaptophysin with EGFP in the mouse retina. (**A**) Structures of the mouse *synaptophysin* locus and the knock-in targeting vector to produce a Syp-EGFP fusion protein. The predicted Cas9-gRNA cutting position is indicated with a scissor symbol. (**B**) Mouse retinas were co-electroporated at P0 with the indicated plasmids. Retinas were harvested at P21, sectioned, and stained with anti-GFP (green) and anti-RFP (red) antibodies and DAPI (blue). Fluorescent signals were predominantly detected in the synaptic terminals of rod photoreceptors (indicated with white arrows), bipolar cells (indicated with yellow arrows), and amacrine cells (indicated with a blue arrow). (**C**,**D**) Tagging endogenous PSD-95 with EGFP in the mouse retina. (**C**) Structures of the mouse *PSD-95* locus and the knock-in targeting vector to produce a PSD-95-EGFP fusion protein. The predicted Cas9-gRNA cutting position is indicated with a scissor symbol. (**D**) Mouse retinas were co-electroporated at P0 with the indicated plasmids. Retinas were harvested at P21, sectioned, and stained with anti-GFP (green) and anti-RFP (red) antibodies, and DAPI (blue). Most of the fluorescent signals were detected in the synaptic terminals of rod photoreceptors (indicated with white arrows). The synaptic terminals of bipolar cells (indicated with a yellow arrow) were also labeled. ONL, outer nuclear layer; INL, inner nuclear layer.

To achieve fluorescent labeling of endogenous Syp and PSD-95 in the mouse retina, the CRISPR gRNAs were designed to target the exons containing the stop codons of the *Syp* and *PSD-95* genes, and the targeting vectors were designed to express Syp-EGFP and PSD-95-EGFP fusion proteins from the endogenous loci (Fig. 5, Supplementary Figs S6, S7). As we found that the use of two distinct gRNAs whose target sequences are adjacent to each other significantly enhances the knock-in efficiency compared to that of a single gRNA (Supplementary Fig. S8), two gRNAs were used together to increase the knock-in efficiency. We observed CRISPR/Cas9-dependent gene knock-in at both gene loci in the retina, with a frequency of ~6% and ~3%, respectively. When endogenous Syp, which is localized in the outer plexiform layer (OPL) and the inner plexiform layer (IPL) of the retina, was labeled with EGFP by *in vivo* gene targeting, fluorescent signals were predominantly detected in the synaptic terminals of rod photoreceptors, bipolar cells, and amacrine cells. Weaker fluorescent signals were also detected in the cell bodies of these retinal neurons (Fig. 5B). Occasionally, fluorescently labeled rod photoreceptor and bipolar cells connected to each other were observed. On the other hand, when endogenous PSD-95, which is predominantly localized in the OPL of the retina, was labeled with EGFP, most of the fluorescent signals were detected in the synaptic terminals of rod photoreceptors (Fig. 5D). The synaptic terminals of bipolar cells in the OPL were also labeled though at much lower frequencies.

### Monitoring protein dynamics of PSD-95 *in vivo*

**Figure 6:**
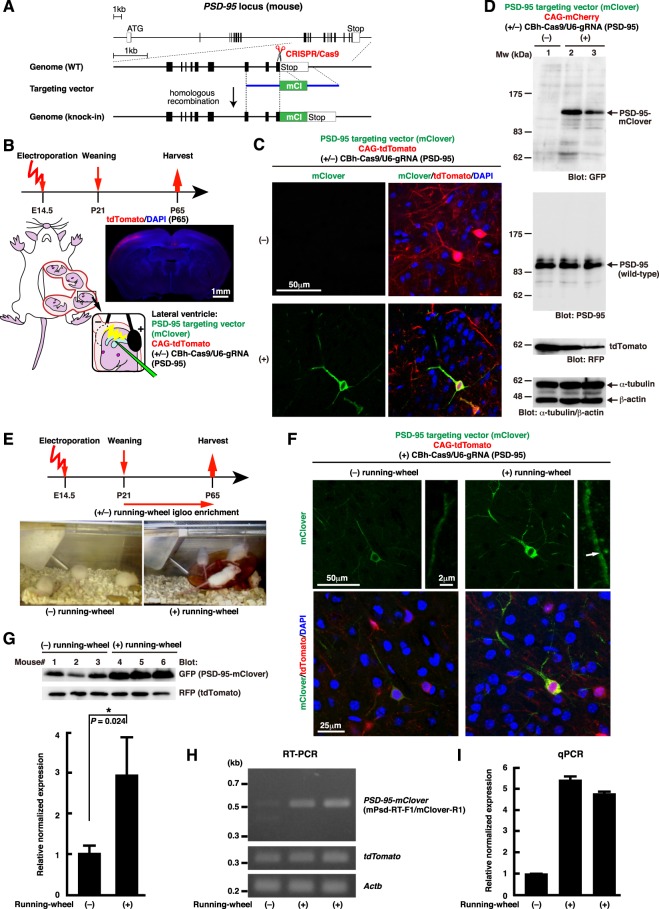
Tagging endogenous PSD-95 with mClover in the developing mouse brain. (**A**) Structures of the mouse *PSD-95* locus and the knock-in targeting vector to produce a PSD-95-mClover fusion protein. The predicted Cas9-gRNA cutting position is indicated with a scissor symbol. (**B**) Experimental design for *in vivo* electroporation to the developing mouse brain. Site-directed electroporation to the somatosensory cortex was revealed by tdTomato fluorescence (Red). Cell nuclei were visualized with DAPI (blue). (**C**) E14.5 mouse brains were co-electroporated with the following plasmids: PSD-95 targeting vector (mClover), CAG-tdTomato, and CBh-Cas9 with (+) or without (−) gRNAs for *PSD-95*. Brains were harvested at P65, sectioned and stained with anti-GFP (green), and anti-RFP (red) antibodies. Cell nuclei were visualized with DAPI (blue). (**D**) Western blot analysis of brains electroporated with the indicated plasmids at E14.5 and harvested at P65. Three independently electroporated brains were analyzed (1 brain per lane), and membranes were probed with indicated antibodies. A PSD-95-mClover fusion protein (~110 kDa) was detected only in the presence of CBh-Cas9/U6-gRNA (PSD-95) (lanes 2, 3). Full-length gel images are presented in Supplementary Fig. S10A. (**E**) Timeline for exposure to running-wheel enrichment. (**F**) E14 mouse brains were co-electroporated with the indicated plasmids, and the electroporated mice were kept in normal cages ((−) running-wheel) or in cages with running wheels ((+) running-wheel). Brains were harvested at P65, sectioned and stained with anti-GFP (green) and anti-RFP (red) antibodies. Cell nuclei were visualized with DAPI (blue). A white arrow indicates a mClover-positive punctate along a dendritic shaft. (**G**) Western blot analysis of brains shown in (**F**). Six independently electroporated brains were analyzed (1 brain per lane), and membranes were probed with indicated antibodies. Full-length gel images are presented in Supplementary Fig. S10B. The amount of PSD-95-mClover was determined by quantifying the intensity of Western blot bands, and relative expression levels of PSD-95-mClover were shown. Data represent mean +/− SD (n = 3; *P* = 0.024, Student’s *t* test). (**H**,**I**) RT-PCR analysis of brains shown in (**F**). Three independently electroporated brains were analyzed (1 brain per lane). PCR primer sets were used to detect cDNAs encoding *PSD-95-mClover*, *tdTomato*, and endogenous mouse *β-actin* (*Actb*), respectively. Full-length gel images are presented in Supplementary Fig. S10C. Relative expression levels of *PSD-95-mClover* determined by qPCR and normalized to those of *tdTomato* were shown in (**I**).

Finally, we examined if *in vivo* gene targeting can be used to label endogenous proteins of interest with fluorescent reporters to monitor their expression changes in specific subpopulations of neurons at a single cell resolution. It is well known that experience-dependent plasticity has been thought to involve increases in the expression of synaptic proteins^33^. But such studies were based on global expression analysis using tissue lysates. Taking advantage of our labeling strategy developed in this study, we tried to label endogenous PSD-95 *in vivo* in the developing mouse brain (Fig. 6, Supplementary Fig. S9).

Embryonic day 14.5 (E14.5) mouse brains were co-electroporated *in utero* with PSD-95 targeting vector (mClover), CAG-tdTomato, and two CRISPR constructs (CBh-Cas9/U6-gRNA (PSD-95)). We could specifically transfect neurons in the somatosensory cortex by site-directed electroporation as revealed by tdTomato fluorescence (Fig. 6B). When the transfected brains were harvested at P65, a PSD-95-mClover fusion protein was detected only in the presence of the CRISPR constructs (Fig. 6C,D), showing that endogenous PSD-95 was correctly tagged with mClover in the mouse brain by *in vivo* gene targeting (Supplementary Fig. S9).

To test whether the expression of endogenous PSD-95 tagged with mClover is increased by experience-dependent plasticity, electroporated mice were separated into two groups at P21. One group was kept in normal cages, and the other group was placed in the enriched cages with running wheels (Fig. 6E). In the mice exposed to the enriched environment, brighter mClover expression was observed at P65, and the density of mClover-positive puncta was also increased (Fig. 6F). Western blot and qPCR analyses showed that PSD-95-mClover protein and mRNA were both up-regulated several-fold by enrichment (Fig. 6G–I). These results show that our *in vivo* genome engineering technique enables quantitative analysis of not only endogenous proteins but also mRNAs in specific subpopulations of neurons at a single cell level.

## Discussion

Our results showed that endogenous proteins, including rhodopsin, glutamine synthetase, arrestin, synaptophysin, and PSD-95 can be efficiently labeled with fluorescent proteins *in vivo* in the mouse retina and brain, by using the combined methods of CRISPR/Cas9 and *in vivo* electroporation. We also showed that using this technique, physiological changes in localization and expression levels of endogenous proteins can be monitored *in situ*. Moreover, we provided a strategy to engineer the mouse genome *in vivo* to express foreign genes from the endogenous gene loci using a self-cleaving 2 A peptide.

Compared with the existing protein-labeling methods, this method is superior for the following reasons. First, this labeling method is easy and rapid. In contrast to the conventional knock-in methods using mouse ES cells, this method significantly shortens the necessary time for tagging of endogenous proteins *in vivo* because of the direct modification of genomic DNA in the internal organ of living mice. Second, this labeling method is accurate. As endogenous proteins are labeled via modification of the genome coding for them, but not via direct chemical modification of their amino acid residues, the specificity of labeling is extremely high. Third, this method is more versatile. In this study, we used the mouse retina and brain as *in vivo* model systems, but the established labeling technique should be widely applicable to endogenous proteins in other organs. We also believe that this method can be used in other animal models besides mice. In addition, since this method does not require the fixation of cells, it is suitable for the observation of the dynamic behavior of labeled proteins in living cells and tissues. Fourth, this labeling method is ideal for imaging of neurons with complicated morphology at a single-cell resolution. As only a limited number of transfected cells undergo CRISPR/Cas9-mediated homologous recombination, this method facilitates “sparse cell labeling”, which is an important approach to study the neural circuit.

During preparation of the manuscript, other groups reported that endogenous proteins in the developing mouse brain can be tagged with the reporters using the approach similar to ours^34–36^. Mikuni *et al*. reported that several endogenous proteins can be labeled with the HA tag in neurons in the developing mouse brain using ~200 base single-stranded oligodeoxynucleotides (ssODNs) as knock-in donors at an efficiency of 0.6–7.5%. They also reported that endogenous CamKIIα and CamKII*β* can be labeled with EGFP using donor plasmids carrying 0.7–0.9 kb homology arms, although the knock-in efficiency was less than 1%^34^. Uemura *et al*. reported that endogenous *β*-actin (Actb) can be tagged with EGFP in neurons in the developing mouse brain using a donor plasmid carrying 1.4 kb and 0.7 kb homology arms at an efficiency of ~1.7%^35^. Tunekawa *et al*. reported that endogenous *β*III-tubulin (Tubb3) in neurons in the developing mouse brain can be tagged with EGFP using a donor plasmid carrying 1 kb and 1.8 kb homology arms at an efficiency of ~20%^36^.

In our study, we showed that endogenous proteins in the developing mouse retina and brain can be labeled with fluorescent reporters at an efficiency of 1-10%. We also found that endogenous proteins labeled with fluorescent reporters can be readily visualized in glial cells as well as in neurons in intact mouse retinas without making sections if the target proteins are relatively abundantly expressed. In addition, we further demonstrated that the dynamics of the tagged endogenous proteins can be easily monitored *in situ*. For example, we observed light-dependent translocation of endogenous arrestin tagged with EGFP within photoreceptors (Fig. 4). We also observed that the expression of endogenous PSD-95 tagged with mClover is increased in mice exposed to the enriched environment at both mRNA and protein levels (Fig. 6). Our current study, together with the other groups’ recent reports, not only presents the versatility of the labeling method among different neural tissues including brain and retina, but also guarantees the accuracy with the “C-terminal fusion approach”.

Although the labeling technique developed in this study is very powerful, there exist several issues that must be overcome for the dissemination and popularization of this technique. The first challenge is that this labeling method is not necessarily suitable for N-terminal tagging of target proteins with fluorescent reporters. In this study, we used the knock-in targeting vectors that correspond to the C-termini of target proteins and do not contain detectable promoter activities. However, when a targeting vector is designed against the genomic DNA encoding the N-terminus (ATG start codon) of protein, there is a high probability that the promoter region is included in the homology arm of the targeting vector^37^. In that case, the targeting vector would express a fluorescent reporter independently of CRISPR/Cas9-mediated homologous recombination, and it becomes hard to distinguish the true signals derived from fluorescent protein-tagged endogenous proteins. For this reason, careful attention is required when one wishes to fuse a fluorescent protein to the N-terminus of a target protein by genome editing.

The “C-terminal fusion approach” is also beneficial to reducing the risk of unintended mutations in the regulatory regions, including enhancers and promoters, of target genes. Cas9 might induce unintended indel mutations mediated by NHEJ in transfected cells if homologous recombination does not occur. This problem matters particularly in the case of N-terminal tagging since altered sequence near the N-terminal region can change the transcription and translation of the target genes. On the other hand, in the case of C-terminal tagging, such a risk is relatively low.

Even with the “C-terminal fusion approach”, however, unintended indel mutations mediated by NHEJ could affect the functions of target genes, especially when target PAM sequences are located within coding sequences. This may cause non-cell-autonomous effects to cells with knock-in alleles. Moreover, for some proteins, C-terminal fusion may abolish normal protein functions. For example, most of the ras superfamily small GTPases have the CAAX motif important for membrane anchoring at their C-termini, and therefore the “C-terminal fusion approach” cannot be used for these proteins.

The second challenge is that off-target effects of the Cas9 nuclease cannot be entirely excluded. When the CRISPR/Cas9-mediated protein tagging technology is applied to the developing or mature mouse tissues wherein cells do not proliferate infinitely, it is difficult to analyze precisely the genomes extracted from the cells that underwent homologous recombination. In this study, overexpression of CRISPR/Cas9 in the mouse retina for one year did not cause any apparent cytotoxicity, suggesting that even if there were some adverse effects on cell functions, it would be minimal. However, it is necessary to consider the experimental conditions carefully, depending on the target sequences. Recently, engineered Cas9 variants, including eSpCas9^38^, SpCas9-HF1^39^ and HypaCas9^40^, with reduced off-target activity have been reported. Use of such Cas9 variants may partly overcome the off-target effects.

The third challenge is that the knock-in efficiency is relatively low. In this study, we found that the use of two adjacent gRNAs targeting one locus increased the knock-in efficiency (Fig. S8) A similar approach was used to increase the knock-in efficiency in mouse fertilized eggs and mouse ES cells^41–43^, although the molecular mechanism underlying the enhanced gene knock-in efficiency by two gRNAs remains unknown. Since simultaneous use of two gRNAs exhibited higher DNA cleavage activity compared to each of the single gRNAs^41,42^, two gRNAs may ensure cleavage of target genome, and thereby enhance the knock-in efficiency.

Recently, it was reported that CRISPR/Cas9-mediated DNA cleavage on both ends of homology arms of a donor plasmid, to which gRNA target sites had been introduced, significantly increased the knock-in efficiency^44^. A combination of two gRNAs and *in vivo* linearization of donor plamids may further enhance the knock-in efficiency.

The fourth challenge that must be circumvented is that fluorescent signal intensity may not be high enough for live cell imaging in cases where the expression levels of target proteins are low. With the development of more sensitive fluorescent signal detection systems and brighter fluorescent proteins, the genome editing-based protein tagging technology will become a more powerful tool for monitoring the expression, localization, and functional dynamics of endogenous proteins *in situ*.

## Experimental Procedures

### Animals

All animal experiments were approved by the Institutional Animal Care and Use Committees at Kyoto University and University of Hyogo. All procedures were performed in full compliance with the Fundamental Guidelines for Proper Conduct of Animal Experiments and Related Activities in Academic Research Institutions issued by the Japanese Ministry of Education, Culture, Sports, Science and Technology. Mice were housed under temperature-controlled conditions (temperature: 24 °C) and maintained on a light/dark cycle (12 h each) with *ad libitum* access to food and water. Timed pregnant ICR mice were purchased from Japan SLC (Shizuoka, Japan).

### CRISPR constructs

The CRISPR target sequences (20-nucleotide sequence followed by a protospacer adjacent motif (PAM) of ‘NGG’) were selected manually, or by using the prediction software^45^ (http://www.broadinstitute.org/rnai/public/analysis-tools/sgrna-design). To make plasmids co-expressing *S*. *pyogenes* Cas9 and gRNA, following synthetic oligonucleotides were annealed and cloned into the BbsI sites of pX330^14^ (Addgene #42230). *Rhodopsin*; 5′-CACCGTAAGCCTGGCCAGAGACTG-3′ and 5′-AAACCAGTCTCTGGCCAGGCTTAC-3′.

*Glul*; 5′-CACCGTTCCAATACAAGAACTAAG-3′ and 5′-AAACCTTAGTTCTTGTATTGGAAC-3′.

*Arrestin*; 5′-CACCGTACTGGAGAGAACACAGAA-3′ and 5′-AAACTTCTGTGTTCTCTCCAGTAC-3′.

*PSD-95*; 5′-CACCGAGGAATCAGAGTCTCTCTC-3′ and 5′-AAACGAGAGAGACTCTGATTCCTC-3′, 5′-CACCGAGGAATCAGAGTCTCTCTC-3′ and 5′-AAACGAGAGAGACTCTGATTCCTC-3′.

*Syp*; 5′-CACCGTACATCTGATTGGAGAAGG-3′ and 5′-AAACCCTTCTCCAATCAGATGTAC-3′, 5′-CACCGTCACCAGATTACATCTGAT-3′ and 5′-AAACATCAGATGTAATCTGGTGAC-3′. To construct Rhodopsin promoter-Cas9/U6-gRNA, pX330 was digested with KpnI and AgeI, and the CBh promoter was replaced with the 2.2 kb bovine rhodopsin promoter^26^.

### Knock-in targeting vectors

Homology arms of knock-in targeting vectors were prepared by genomic PCR using mouse genomic DNA extracted from an adult ICR mouse liver as a template. Knock-in targeting vectors were constructed using a conventional cloning technique with restriction enzymes or using the In-Fusion HD cloning kit (Takara Bio, Shiga, Japan). The CRISPR target sequences were removed from the knock-in vectors by introducing silent mutations or deletions into the homology arms. For detail, see Supplemental Experimental Procedures.

### *In vivo* electroporation

*In vivo* electroporation to P0 ICR mouse retina was performed as described^28,46^. Briefly, a DNA mixture (3 μg/μl) composed of a pX330-based CRISPR construct (1 μg/μl), a knock-in targeting vector (1 μg/μl), and a transfection control plasmid (pCAG-EGFP, pCAG-H2B-EGFP pCAG-mCherry, or pCAG-H2B-mCherry, 1 μg/μl) was injected into the subretinal space of P0 mouse pups using a pulled glass needle, and electric pulses (80 V) were applied (five square pulses of 50 ms duration with 950 ms intervals) using the CUY21 electroporator (Nepagene, Chiba, Japan) and electrodes (Nepagene, CUY650-5). When two pX330-based CRISPR constructs were used together, each concentration was 0.5 μg/μl (total 1 μg/μl). *In vivo* electroporation to E14.5 ICR mouse brain was performed as described^47,48^. Briefly, a DNA mixture composed of two pX330-based CRISPR constructs (0.5 μg/μl each), a knock-in targeting vector (1 μg/μl), and pCAG-tdTomato (1 μg/μl) was injected into the lateral ventricles of E14.5 mouse embryos *in utero* using a 35-gauge NanoFil needle (World Precision Instruments) connected to a NanoFil 10 μl syringe (World Precision Instruments), and electric pulses (50 V) were applied (five square pulses of 50 ms duration with 950 ms intervals) using the CUY21 electroporator and electrodes (Nepagene, CUY650-5).

### Housing mice in an enriched environment

The expression of tdTomato in the brains of electroporated mice was checked after the birth using a handheld LED light (OPTOCORD Co. Tokyo, Japan, LED-EXHD/RFP). Only the pups strongly positive for tdTomato in the somatosensory cortex were used in this study. After weaning at P21, mice were separated into two groups, and five mice were maintained per cage (17 cm × 27 cm × 12 cm). One group was put in regular cages, and the other group was in the cages with a running wheel (FAST-Trac, Mouse Igloo, Bio-Serv).

### FACS

For fluorescence cell sorting of mouse retinas, harvested mouse retinas were dissociated into single cells using Neural Tissue Dissociation Kit (Miltenyi Biotech, 130-094-802), resuspended in DMEM/F-12 (Thermo Fisher, 11039-021) containing 5% fetal calf serum (Equitech-Bio), and sorted or analyzed by FACS Aria II (Beckton Dickinson). For single-cell genotyping, sorted cells were collected at one cell per well in a 96-well PCR plate or 8-strip PCR tubes. Each well had been preloaded with 15 μl of 50 μg/ml proteinase K (Roche, 03115879001) in H_2_O. After cell sorting, PCR plates and 8-strip PCR tubes were briefly centrifuged and stored at −80 °C until use.

### Genotyping

For single-cell genotyping, FACS-purified single cells in 15 μl of 50 μg/ml proteinase K were incubated for 1 h at 50 °C, followed by 4 min at 99 °C. Then, nested PCRs were performed using PrimeSTAR GLX DNA polymerase (Takara Bio, Shiga, Japan) or KOD FX neo DNA polymerase (Toyobo, Osaka, Japan). For genotyping of retinal and brain cells transfected with PSD-95 targeting vector (EGFP), PSD95 targeting vector (mClover), or Syp targeting vector (EGFP), genomic DNAs were purified from the harvested mouse retinas or brains using QIAamp DNA mini kit (Qiagen). Then, nested PCRs were performed to detect the knock-in alleles. For detail, see Supplemental Experimental Procedures.

### Immunohistochemistry

Immunostaining of retinal and brain cryosections was performed as described previously^49,50^. Primary antibodies used in this study are as follows: mouse anti-rhodopsin (1:1000, Millipore, MABN15), mouse anti-glutamine synthetase (1:500, Millipore, MAB302), rabbit-anti-PSD-95 (1:500, Cell Signaling Technology, 3450), rabbit anti-synaptophysin (1:500, Cell Signaling Technology, 5461), rabbit anti-Ki67 (1:500, Abcam, ab15580), rabbit anti-phospho-histone H3 (Ser10) (1:500, Cell Signaling Technology, 9701), rabbit anti-GFP (1:500, Thermo Fisher, A11122), goat anti-GFP (1:1000, Sicgen, AB0020), rabbit anti-DsRed (1:500, Takara Bio, Shiga, Japan, 632496), and rat-anti-mCherry (1:500, Thermo Fisher, 11217). Secondary antibodies used in this study include Alexa 488- 594-, or 647-conjugated goat anti-mouse, rat or rabbit IgG (1:500, Thermo Fisher), and Alexa 488- 594-, or 647-conjugated donkey anti-mouse, rat, rabbit, or goat IgG (1:500, Thermo Fisher). Fluorescent images of retinal sections were taken using a confocal microscope (Zeiss LSM 510 META) with a 40 x NA 1.20 C-Apochromat water immersion objective (Zeiss) or a 20 x NA 0.8 Plan-Apochromat objective (Zeiss). Confocal images of cortical brain sections were acquired using a laser-scanning confocal imaging system (FLUOVIEW FV1000-D; Olympus) and a microscope equipped with spectral system (IX81-S; Olympus) and a 100 x NA 1.40 oil objective (Olympus; seven planes with 0.42-μm step width per stack). Images of the intact retina and whole-brain sections were obtained using a stereofluorescence microscope system (M205 FA; Leica) equipped with a CCD camera (DFC365 FX; Leica) and a 1 x NA 0.175 Plan Apo objective (Leica). The images were arranged and labeled using Photoshop (Adobe).

### Western blot

Western blot analysis and preparation of tissue homogenates were performed as described previously^50^. Proteins were separated by SDS–PAGE and were electrophoretically transferred onto polyvinylidene fluoride membrane (Millipore). The membrane was blocked with 3% low-fat milk (Nacalai Tesque, Kyoto, Japan) in Tris-buffered saline (TBS) and then incubated with primary antibodies diluted in 3% low-fat milk in TBS or Can Get Signal immunoreaction enhancer solution (Toyobo, Osaka, Japan) for 16 h at 4 °C. The primary antibodies were detected with horseradish peroxidase (HRP)-conjugated secondary antibodies and chemiluminescence detection kits (Chemi-Lumi One; Nacalai Tesque, Kyoto, Japan). Images were captured using an ImageQuant LAS4000 mini analyzer (GE Healthcare). Primary antibodies used in immunoblotting are as follows: mouse monoclonal antibodies against α-tubulin and *β*-actin (Sigma-Aldrich), a mouse monoclonal antibody against GFP (Santa Cruz Biotechnology, B2), mouse monoclonal antibodies against rhodopsin and glutamine synthetase (Millipore), a rabbit polyclonal antibody against RFP (MBL, PM005), a rabbit polyclonal antibody against GFP (Thermo Fisher, A11122), a rabbit monoclonal antibody against PSD-95 (Abcam, ab76115), a rabbit polyclonal antibody against arrestin (Cell Signaling Technology, 11828). Goat polyclonal secondary antibodies conjugated to HRP were purchased from Dako-Cytomation.

## Supplementary information

Supplementary Information
