# Supplementary material for: Optimized CRISPR/Cas9-mediated in vivo genome engineering applicable to monitoring dynamics of endogenous proteins in the mouse neural tissues
Source: Sci Rep. 2019 Aug 5;9:11309. doi: 10.1038/s41598-019-47721-4 (PMC6683140; doi:10.1038/s41598-019-47721-4)
Supplement: Supplementary file 1 — Supplementary Information [file 41598_2019_47721_MOESM1_ESM.pdf]

Supplementary Information

Optimized CRISPR/Cas9-mediated *in vivo* genome engineering applicable to monitoring dynamics of endogenous proteins in the mouse neural tissues

Takahiko Matsuda and Izumi Oinuma

Supplemental Experimental Procedures

Expression vectors

pCAG-EGFP (Addgene #11150), pRho-DsRed (Addgene #11156), pCALNL-EGFP (Addgene #13770), and pRho-Cre (Addgene #13779) was reported previously[1,2]. pCAG-tdTomato was constructed by digesting pCAG-EGFP with KpnI and NotI, and replacing the coding region of EGFP with that of tdTomato excised from pUB-tdTomato[3] using KpnI and NotI. pCAG-mCherry was constructed by inserting the coding region of mCherry excised from pmCherry-N1 (Takara Bio, Shiga, Japan) with EcoRI and NotI into pCAGEN (Addgene #11160) digested with EcoRI and NotI. pCAG-mClover was constructed by inserting the coding region of monomeric Clover (mClover)[4] with a 5’ linker sequence (5’-GAATTCTGCAGTCGACGGTACCGCGGGCCCGGGATCCACCGGTCGCCACC-3’) synthesized by Thermo Fisher, into the EcoRI and NotI sites of pCAGEN. To construct pCAG-H2BGFP, the cDNA encoding a fusion protein between human histone H2B and EGFP[5] was amplified by PCR using pFMHGW[6] as a template, and primers (5’-AACTCGAGTGAGCCGCCACCATGCCAGAGCCAGCGAAGTCTGCTC-3’ and 5’-GGAGTGCGGCCGCTTTACTTGTACAGCTCG-3’). The amplified PCR fragment was digested with XhoI and NotI, and cloned into pCAGEN digested with XhoI and NotI. pCAG-H2B-mCherry was constructed by digesting pCAG-H2B-EGFP with AgeI and NotI, and replacing the coding region of EGFP with that of mCherry excised from pCAG-mCherry using AgeI and NotI. To construct pRhodopsin promoter-Cas9/U6-gRNA, pX330 was digested with KpnI and AgeI, and the CBh promoter was replaced with the 2.2kb bovine rhodopsin promoter[7] amplified by PCR using primers (5’-ATATGGTACCACGTTGTCAGAAGCGTTAGCGG-3’ and 5’-AGCTACCGGTCGAAGCTTGAGCTCGACTCTAG-3’) and pRho-DsRed as a template, and digested with KpnI and AgeI. To construct pRhodopsin promoter-tdTomato, pRho-DsRed was digested with EcoRI and NotI, and the coding region of DsRed was replaced with that of tdTomato excised from pCAG-tdTomato. To construct pCALNL-tdTomato, pCALNL-EGFP was digested with EcoRI and NotI, and

the coding region of EGFP was replaced with that of tdTomato excised from pCAG-tdTomato. To construct pCAG-PSD-95-EGFP, the coding region of mouse *PSD-95* was amplified by RT-PCR using primers (5’-ATATCTCGAGCTGAGCCGCCACCATGGACTGTCTCTGTATAGTGACAAC-3’ and 5’-AATTACCGGTGGATCCGAGTCTCTCTCGGGCTGGGACCCAGAT-3’) and total RNA extracted from adult ICR mouse retina. Linker-EGFP sequence was generated by PCR using primers (5’-CTCGGATCCACCGGTAATTCCGCTGACGGCGGCGGAGGATCGGGTGGTAGTGGTGGTTCAGGAGGAGGATCGACCCAAGGAGGTACCGTGAGCAAGGGCGAGGAGCTGTT-3’ and 5’-ATGCGCGGCCGCTTTACTTGTACAGCTC-3’) and pCAG-EGFP as a template. These PCR fragments were digested with XhoI and AgeI, AgeI and NotI, respectively, and simultaneously cloned into pCAGEN digested with XhoI and NotI. Restriction sites in primer sequences were underlined.

Knock-in targeting vectors

Homology arms of knock-in targeting vectors were prepared by genomic PCR using mouse genomic DNA extracted from an adult ICR mouse liver as a template. The CRISPR target sequences were removed from the knock-in vectors by introducing silent mutations or deletions into the homology arms.

Rhodopsin targeting vector (EGFP): To construct a cloning plasmid vector, the pUC plasmid backbone was amplified by PCR using primers (5’-*TGTATCTAAGCTTAATTA*AGATCGCGTTTAAACGGCGCGCCGCTGCATTAATGAATCGGCCA-3’ and 5’-*CCGCGGTACCGTCGAC*ATTTAAATGGCCGGCCCAGGTGG-3’) and pCAG-EGFP as a template. The coding region of EGFP was amplified by PCR using primers (5’-*GTCGACGGTACCGCGG*GCCCGG-3’ and 5’-GATTATGAGCGGCCGCTTTACTTGTACAGCTCGTCCAT-3’) and pCAG-EGFP as a template. SV40 polyA sequence was amplified by PCR using primers (5’-*AAGCGGCCGCTCATAATC*AGCCATACCACATTTGTA-3’ and 5’-TAATTAAGCTT*AGATACATTGATGAG*TTTGGACA-3’) and pEGFP-N1 (Takara Bio, Shiga, Japan) as a template. These PCR fragments were simultaneously ligated using the In-Fusion HD Cloning Kit (Takara Bio, Shiga, Japan) to generate pUC-EGFP-pA. The 5’ homology arm (1033 bp) was amplified by PCR using primers (5’-ACGTATTTAAATGGGACAGGGGAGGCATTGCACTC-3’ and 5’-CGGTGGATCCCCGGCTGGAGCCACCTGGCTGGTC-3’). The 3’ homology arm

(1114 bp) was amplified by PCR using primers (5’-ATATGCGGCCGCGCTGAAAGTAGGAGTCTCCTGTCCCCA-3’ and 5’-CGATGTTTAAACTCCATACTGGCCTGGAACTG-3’). The 5’ homology arm digested with SwaI and BamHI was ligated into pUC-EGFP-pA digested with SwaI and BamHI to generate pUC-(Rho 5’ 1033 bp)-EGFP-pA. Then the 3’ homology arm digested with NotI and PmeI was ligated into pUC-(Rho 5’ 1033 bp)-EGFP-pA digested with NotI and PmeI to generated Rhodopsin targeting vector (EGFP).

Rhodopsin targeting vector (mCherry): Rhodopsin targeting vector (EGFP) was digested with AgeI and NotI, and the coding region of EGFP was replaced with that of mCherry excised from pCAG-mCherry with AgeI and NotI.

Rhodopsin targeting vector (2A-EGFP-pA): The 5’ homology arm (1031 bp) was amplified by PCR using primers (5’-ACGTATTTAAATGGGACAGGGGAGGCATTGCACTC-3’ and 5’-ATCTGGTACCGGCTGGAGCCACCTGGCTGGTC-3’) and Rhodopsin targeting vector (EGFP) as a template. 2A-EGFP was generated by PCR using primers (5’-AGCCGGTACCAGATCTGAGGGCAGAGGAAGTCTTCTAACATGCGGTGACGTGGAGGAGAATCCCGGCCCTATGGTGAGCAAGGGCGAGGAGCT-3’ and 5’-ATGAGCGGCCGCTTTACTTGTACAGCTCGTCCATG-3’) and pCAG-EGFP as a template. These PCR fragments were digested with SwaI and KpnI, KpnI and NotI, respectively, and simultaneously ligated into pUC-EGFP-pA digested with SwaI and NotI to generate pUC-(Rho 5’ 1033 bp)-2A-EGFP-pA. Then the 3’ homology arm (1114 bp) was amplified by PCR using primers (5’-ATCTAAGCTTGCTGAAAGTAGGAGTCTCCTGTC-3’ and 5’-CGATGTTTAAACTCCATACTGGCCTGGAACTG-3’) and Rhodopsin targeting vector (EGFP) as a template, digested with HindIII and PmeI, and ligated into pUC-(Rho 5’ 1033 bp)-2A-EGFP-pA digested with HindIII and PmeI, to generate Rhodopsin targeting vector (2A-EGFP-pA).

Rhodopsin targeting vector (2A-H2B-EGFP): The 5’ homology arm (1031 bp) was amplified by PCR using primers (5’-ACGTATTTAAATGGGACAGGGGAGGCATTGCACTC-3’ and 5’-ATCTGGTACCGGCTGGAGCCACCTGGCTGGTC-3’) and Rhodopsin targeting vector (EGFP) as a template. 2A-H2B-EGFP was generated by PCR using primers (5’-AGCCGGTACCAGATCTGAGGGCAGAGGAAGTCTTCTAACATGCGGTGACGTGGAGGAGAATCCCGGCCCTATGCCAGAGCCAGCGAAGTCTGC-3’ and 5’-ATGAGCGGCCGCTTTACTTGTACAGCTCGTCCATG-3’) and pCAG-H2B-EGFP as

a template. These PCR fragments were digested with SwaI and KpnI, KpnI and NotI, respectively, and simultaneously ligated into Rhodopsin targeting vector (EGFP) digested with SwaI and NotI to generate Rhodopsin targeting vector (2A-H2B-EGFP).

Rhodopsin targeting vector (2A-H2B-EGFP) deletion constructs: To delete 5’- and 3’-homology arms of Rhodopsin targeting vector (2A-H2B-EGFP), PCRs were performed using Rhodopsin targeting vector (2A-H2B-EGFP) as a template, and following primers. 5’_Fw_0.6kb: 5’-ATATCTCGAGTCCAAGTCTGGGAATAGAATGGTC-3’. 5’_Fw_0.3kb: 5’-ATATCTCGAGGACACATGGAATGCCACTTGAC-3’. 5’_Fw_0.2kb: 5’-ATATCTCGAGCCAGGCTTAGTGAGGGGACATGC-3’. 3’_Rv_0.5kb: 5’-ATCGAGATCTCAGAGAAAACCAGGGCAGGTATGT-3’. 3’_Rv_0.2kb: 5’-ATCGAGATCTGGTTGAGGATGGTCTTGGTGGAT-3’. 3’_Rv_0.1kb: 5’-ATCGAGATCTAGAACACTGAGGGAGCCTGCATG-3’. Amplified PCR fragments were digested with XhoI and BglII, and ligated into the pUC plasmid backbone amplified by PCR using primers (5’-ATATAGATCTGCTGCATTAATGAATCGGCCAA-3’ and 5’-CGATCTCGAGGTGGCACTTTTCGGGGAAATGTGC-3’) and pCAG-EGFP as a template and digested with XhoI and BglII, or pBluescript II KS(-) (Agilent) digested with XhoI and BamHI.

GluI targeting vector (mClover): The 5’ homology arm (1143 bp) was amplified by PCR using primers (5’-ATATCTCGAGTATTACTGCGGTGTGGGAGCAGAC-3’ and 5’-ATCTGGTACCTTCTTATACTGAAAGGGTTCGTCGCCT-3’). The 3’ homology arm (978 bp) was amplified by PCR using primers (5’-ATATGCGGCCGCAGACTTCCAGTGATCCCTCTCCCAG-3’ and 5’-ATCGAGATCTCCATCAGTAACAGTGTTCAAGTTG-3’). Amplified PCR fragments were digested with XhoI and KpnI, NotI and BglII, respectively. The coding sequence of mClover was excised from pCAG-mClover with KpnI and NotI. These three fragments were simultaneously ligated into the pUC plasmid backbone amplified by PCR using primers (5’-ATATAGATCTGCTGCATTAATGAATCGGCCAA-3’ and 5’-CGATCTCGAGGTGGCACTTTTCGGGGAAATGTGC-3’) and pCAG-EGFP as a template, and digested with XhoI and BglII, to generate GluI targeting vector (mClover).

Arrestin targeting vector (EGFP): The genomic DNA including exon 16 of *arrestin* was amplified by PCR using primers (5’-*CCACCTCGAGTAAGTCATCT*GGAAGCTTCTCTGGG-3’ and 5’-*AATGAGATCTTCTGGACACTA*TGGCTAGGAATGG-3’). The pUC plasmid

backbone was amplified by PCR using primers (5’-*TAGTGTCCAGAAGATCTCATT*AATGAATCGGCCAACG-3’ and 5’-*AGATGACTTACTCGAGGTGG*CACTTTTCGGGGAAATGTGC-3’) and pCAG-EGFP as a template. These two PCR fragments were ligated using the In-Fusion HD Cloning Kit (Takara Bio, Shiga, Japan) to generate pUC-arrestin genomic DNA. Then two PCRs were performed using primers (5’-*TCAGTATTTTCACCT*GTATCTTTCAGATTTTGGCGAG-3’ and 5’-*GAAACTGCTTGTAGTT*GAAGCCCCCGGTAGACTCTCA-3’) and pUC-arrestin genomic DNA as a template, and primers (5’-*AGGTGAAAATACTGA*AGGTAAGAAAGATGAGGATGCTGGCCAGGATGAGGGATCCACCGGTAATTCCGCTGACG-3’ and 5’-*AACTACAAGCAGTTTC*TAGGCATCTAGGCTAAGTCTGCGGCCGCTTTACTTGTACAGC-3’) and pCAG-PSD-95-EGFP as a template, respectively. The two PCR fragments were ligated using the In-Fusion HD Cloning Kit to generate Arrestin targeting vector (EGFP) that contains the 899 bp 5’ homology arm and the 997 bp 3’ homology arm.

PSD-95 targeting vector (mClover): The genomic DNA including exon 20 of *PSD-95* was amplified by PCR using primers (5’-ATATGTCGACTGCATCCTTGATGTCTCAGCCAATG-3’ and 5’-AGTCAAGCTTTCTGTCTGCCTCGGTCTGGTAAGTC-3’). The PCR fragment was digested with SalI and HindIII, and ligated into the pUC plasmid backbone prepared from pUC-EGFP-pA digested with SalI and HindIII, to generate pUC-PSD-95 genomic DNA1. Then PCR was performed using primers (5’-*AAATGCTAAGCGGCCGC*CTGGACTCACCCTGCCTCCAC-3’ -3’ and 5’-*AATTACCGGTGGATCC*GAGTCTCTCTCGGGCTGGGAC-3’) and pUC-PSD-95 genomic DNA1 as a template. The PCR fragment was ligated with linker-mClover prepared by PCR using primers (5’-*GATCCACCGGTAATT*CCGCTGACGGCGGCGGAGGATCGGGTGGTAGTGGTGGTTCAGGAGGAGGATCGACCCAAGGAGGTACCGTGAGCAAGGGCGAGGAGCTG-3’ and 5’-*GCGGCCGCTTAGCATTTA*GGTGA-3’) and pCAG-mClover as a template, using the In-Fusion HD Cloning Kit, to generate PSD-95 targeting vector (mClover) that contains the 1037 bp 5’ homology arm and the 966 bp 3’ homology arm.

PSD-95 targeting vector (EGFP): The genomic DNA including exon 20 of *PSD-95* was amplified by PCR using primers (5’-*CCTGGTCGACAATGCAGCCT*GCCAGGAAGGAGGTA-3’ and

5’-*CAGCAAGCTTAGCAAGT*GGCTTATCTGTCAAGTT-3’). The pUC plasmid backbone was amplified by PCR using primers (5’-*ACTTGCTAAGCTTGCTG*CATTAATGAATCGGCCAACG-3’ and 5’-*AGGCTGCATTGTCGACCAGG*TGGCACTTTTCGGGGAAATG-3’) and pCAG-EGFP as a template. These two PCR fragments were ligated using the In-Fusion HD Cloning Kit to generate pUC-PSD-95 genomic DNA2. Then PCR was performed using primers (5’-*TACAAGTAAAGCGGCCGC*TGGACTCACCCTGCCTCCAC-3’ and 5’-*AATTACCGGTGGATCC*GAGTCTCTCTCGGGCTGGGAC-3’) and pUC-PSD-95 genomic DNA2 as a template. The PCR fragment was ligated with linker-EGFP excised from pCAG-PSD-95-EGFP with BamHI and NotI, using the In-Fusion HD Cloning Kit to generate PSD-95 targeting vector (EGFP) that contains the 496 bp 5’ homology arm and the 2039 bp 3’ homology arm.

Syp targeting vector (mClover): The genomic DNA including exon 6 of *Syp* was amplified by PCR using primers (5’-ATATGTCGACTTGAGACAGGATCTACTTATGTGAC-3’ and 5’-AGTCAAGCTTCACACACAGGGTCTCCAAGCAGCCT-3’). The PCR fragment was digested with SalI and HindIII, and ligated into the pUC plasmid backbone prepared from pUC-EGFP-pA digested with SalI and HindIII, to generate pUC-Syp genomic DNA1. Then PCR was performed using primers (5’-*AAATGCTAAGCGGCCGC*TAATCTGGTGAGTGACAACTG-3’ and 5’-*AATTACCGGTGGATCC*GGAGAAGGAGGTGGGCGCACC-3’) and pUC-Syp genomic DNA1 as a template. The PCR fragment was ligated with linker-mClover prepared by PCR using primers (5’-*GATCCACCGGTAATT*CCGCTGACGGCGGCGGAGGATCGGGTGGTAGTGGTGGTTCAGGAGGAGGATCGACCCAAGGAGGTACCGTGAGCAAGGGCGAGGAGCTG-3’ and 5’-*GCGGCCGCTTAGCATTTA*GGTGA-3’) and pCAG-mClover as a template, using the In-Fusion HD Cloning Kit, to generate Syp targeting vector (mClover) that contains the 894 bp 5’ homology arm and the 964 bp 3’ homology arm.

Syp targeting vector (EGFP): The genomic DNA including exon 6 of *Syp* was amplified by PCR using primers (5’-*CCTGGTCGACATGCCCATTC*CTGTTCACTCAGCC-3’ and 5’-*CAGCAAGCTTACCTTTC*TTCTGGACTCCATGGACA-3’). The pUC plasmid backbone was amplified by PCR using primers (5’-*GAAAGGTAAGCTTGCTG*CATTAATGAATCGGCCAACG-3’ and

5'-*GAATGGGCATGTCGACCAGG*TGGCACTTTTCGGGGAAATG-3') and pCAG-EGFP as a template. These two PCR fragments were ligated using the In-Fusion HD Cloning Kit to generate pUC-Syp genomic DNA 2. Then PCR was performed using primers (5'-*TACAAGTAAAGCGGCCGC*TAATCTGGTGAGTGACAACTG-3' and 5'-*AATTACCGGTGGATCC*GGAGAAGGAGGTGGGCGCACC-3') and pUC-Syp genomic DNA 2 as a template. The PCR fragment was ligated with linker-EGFP excised from pCAG-PSD-95-EGFP with BamHI and NotI, using the In-Fusion HD Cloning Kit to generate Syp targeting vector (EGFP) that contains the 405 bp 5' homology arm and the 2275 bp 3' homology arm. Restriction sites in primer sequences were underlined, and homology overlaps in primer sequences for In-Fusion cloning were shown in italics.

.

Single-strand annealing (SSA) assay

Activities of CRISPR constructs were evaluated in HEK293T cells using a single-strand annealing assay as described previously[8]. The reporter plasmid (pCAG-split Luc2) encoding two split inactive parts of the luciferase gene with overlapped sequences was constructed as follows. Luc2 (1-1131) was amplified by PCR using primers (5'-ATGCGAATTCTGAGCCGCCACCATGGAAGATGCCAA-3' and 5'-CTCCTACTCGAGGTAACGCAACTAAGTCATAGTCCGCCTAGAAGCATTTGCGGTGGACGATGGAGGGTACCCTAGTCCAAGTCCACCACCTTAGC-3') and pGL4.10 (Promega) as a template. Luc2 (331-1635) was amplified by PCR using primers (5'-CGTTACCTCGAGTAGGAGCGCGAGCTGCTGAACAGC-3' and 5'-ATATGCGGCCGCTTACACGGCGATCTTGCCGCCCTTCTTG-3') and pGL4.10 as a template. These PCR fragments were digested with EcoRI and XhoI, and XhoI and NotI, respectively, and simultaneously cloned into pCAGEN digested with EcoRI and NotI. Mouse genomic sequences containing CRISPR target sites were amplified by PCR using following primers.

Rhodopsin; 5'- ATATGGTACCAGATGACGACGCCTCTGCCACC-3' and 5'-ATATCTCGAGCAGGAGACTCCTACTTTCAGCC-3'.

Glul; 5'-ATATGGTACCTGCCGGTGTTGCCAACCGCGGTGC-3' and 5'-ATGCCTCGAGTGGTGTGTAGAGATTAAGAACCTG-3'.

Arrestin; 5'- ATATCTCGAGTCCTCAGCTTCTGAGAGTAAGCACTG-3' and 5'-ATATGGTACCTGACACATGATAAGAGGACCCAGAG-3'.

PSD-95; 5'- ATCGCTCGAGAGAGATCAATAAGCGGATCACAGA-3' and 5'-ATGCGGTACCCCACAGTTAGACCTTCCACTCATG-3'.

Syp; 5'- ATCGCTCGAGGTGTTTGGCTTCCTGAACCTGGTG-3' and 5'-ATGCGGTACCTCCTCCACCTACTGGCGTTACCTCC-3'. Adult ICR mouse genomic DNA was used as a PCR template. Amplified PCR fragments were digested with KpnI and XhoI, and inserted between Luc2 (1-1131) and Luc2 (331-1635) of pCAG-split Luc2 digested with KpnI and XhoI. HEK293T cells were maintained in DMEM (Thermo Fisher, 11965) supplemented with 10% FCS (Equitech-Bio) and antibiotics (100 u/ml penicillin and 100 μg/ml streptomycin, Nacalai Tesque, Kyoto, Japan). Cells were plated onto 24-well dishes at a density of $1x10^5$ cells per well. Eighteen hours later, the cells were co-transfected with 50 ng of the pCAG-split Luc2 reporter construct and 100 ng of the CRISPR construct using polyethylenimine (Polysciences, 24765-2). Twenty-four hours after transfection, the cells were harvested, and luciferase activity was measured using Luciferase Assay System (Promega, E1500) and Lumat LB 9507 (Berthold Technologies). Restriction sites in primer sequences were underlined.

Genotyping

Single-cell genotyping was performed by nested PCR using PrimeSTAR GLX DNA polymerase (Takara Bio, Shiga, Japan) or KOD FX neo DNA polymerase (Toyobo, Osaka, Japan). FACS-purified single cells in 15 μl of 50 μg/ml proteinase K were incubated for 1 h at 50°C, followed by 4 min at 99°C. For single-cell genotyping of retinal cells transfected with Rhodopsin targeting vector (EGFP), the first PCR was carried out in a reaction volume of 50 μl with four primers: Primers (5'-CATCTATAACCCGGTCATCTACATC-3' and 5'-GTGCAGATGAACTTCAGGGTCAGCTTG-3') to amplify the EGFP knock-in allele (1610 bp) and primers (5'-TCATGTTTGAGACCTTCAACACC-3' and 5'-TCCTGCTCGAAGTCTAGAGCAAC-3') to amplify *Actb* (313 bp) as a positive control. Reaction conditions were: 1 cycle of 98°C for 3 min, 30 cycles of 98°C for 10 sec, 58°C for 30 sec and 68°C for 2 min. Then, using 0.5 μl of the first PCR products as a template, the second PCR was carried out in a reaction volume of 25 μl with primers (5'-CATCTATAACCCGGTCATCTACATC-3' and 5'-GTGCAGATGAACTTCAGGGTCAGCTTG-3') to amplify the EGFP knock-in allele (1168 bp) or primers (5'-ATGTACGTAGCCATCCAGGCTGT-3' and 5'-AGCTGTGGTGGTGAAGCTGTAG-3') to amplify *Actb* (219 bp). Reaction

conditions were: 1 cycle of 98°C for 3min, 30 cycles of 98°C for 10 sec, 60°C for 15 sec and 68°C for 1 min. For single-cell genotyping of retinal cells transfected with Glul targeting vector (mClover), the first PCR was carried out in a reaction volume of 50 μl with primers (5’-GCAGGACTAGTGACTCAGGAAAGCAGG-3’ and 5’-CTGAACTTGTGGCCGTTTACGTC-3’) to amplify the mClover knock-in allele (1393 bp) and the primers to amplify *Actb*. Then, using 0.5 μl of the first PCR products as a template, the second PCR was carried out in a reaction volume of 25 μl with primers (5’-TGCTGTGTCTTGAACCTCCTTCA-3’ and 5’-CGTCCAGCTCGACCAGGATGGGCAC-3’) to amplify the mClover knock-in allele (1278 bp). For single-cell genotyping of retinal cells transfected with Arrestin targeting vector (EGFP), the first PCR was carried out in a reaction volume of 50 μl with primers (5’-ACAACCATCTGTAATAGAATCCGATGC-3’ and 5’-GTGCAGATGAACTTCAGGGTCAGCTTG-3’) to amplify the EGFP knock-in allele (1249 bp) and the primers to amplify *Actb*. Then, using 0.5 μl of the first PCR products as a template, the second PCR was carried out in a reaction volume of 25 μl with primers (5’-GTACAGCTGCAGTGTATTCACACATA-3’ and 5’-GACACGCTGAACTTGTGGCCGTTTACG-3’) to amplify the EGFP knock-in allele (1202 bp).

For genotyping of retinal and brain cells transfected with PSD-95 targeting vector (EGFP), PSD95 targeting vector (mClover), Syp targeting vector (EGFP), or Syp targeting vector (mClover), genomic DNAs were purified from the harvested mouse retinas or brains using QIAamp DNA mini kit (Qiagen). Nested PCRs were performed to detect the knock-in alleles. The first PCRs were carried out in a reaction volume of 25 μl: 1 cycle of 98°C for 4 min, 20 cycles of 98°C for 10 sec, 60°C for 15 sec and 68°C for 1 min. Using 0.5 μl of the first PCR products as a template, the second PCRs were carried out in a reaction volume of 25 μl: 1 cycle of 98°C for 3 min, 27 cycles of 98°C for 10 sec, 60°C for 10 sec and 68°C for 40 sec.

For genotyping of retinal cells transfected with PSD95 targeting vector (EGFP), the first PCR was performed with primers (5’-ATCTGTCACTTTCC CTTTGGCCAAC-3’ and 5’-GTGCAGATGAACTTCAGGGTCAGCTTG-3’, product size 1409 bp) and the second PCR was performed with primers (5’-ATATTCTCTGTCTCTTCCTGGCACC-3’ and 5’-GACACGCTGAACTTGTGGCCGTTTACG-3’, product size 1308 bp). For genotyping of retinal cells transfected with PSD95 targeting vector (mClover), the first PCR was performed with primers (5’-ATCTGTCACTTTCC CTTTGGCCAAC-3’ and 5’-CTGAACTTGTGGCCGTTTACGTC-3’, product size 1329 bp) and the second PCR

was performed with primers (5’-ATATTCTCTGTCTCTTCCTGGCACC-3’ and 5’-CGTCCAGCTCGACCAGGATGGGCAC-3’, product size 1260 bp). For genotyping of retinal cells transfected with Syp targeting vector (EGFP), the first PCR was performed with primers (5’-CACCACTTGAGAAGCAGAAGCATGTAG-3’ and 5’-GTGCAGATGAACTTCAGGGTCAGCTTG-3’, product size 1534 bp) and the second PCR was performed with primers (5’-TATATATGCATCAATGAGTGATGTCTG-3’ and 5’-GACACGCTGAACTTGTGGCCGTTTACG-3’, product size 1297 bp).
For genotyping of retinal cells transfected with Syp targeting vector (mClover), the first PCR was performed with primers (5’-CACCACTTGAGAAGCAGAAGCATGTAG-3’ and 5’-CTGAACTTGTGGCCGTTTACGTC-3’, product size 1441 bp) and the second PCR was performed with primers (5’-TATATATGCATCAATGAGTGATGTCTG-3’ and 5’-CGTCCAGCTCGACCAGGATGGGCAC-3’, product size 1266 bp).
Electroporated pCAG-mCherry was detected by PCR using primers (5’-TAACATGGCCATCATCAAGGAGTTC-3’ and 5’-AGCCCATGGTCTTCTTCTGCATTAC-3’, product size 419 bp), and the following condition: 1 cycle of 98°C for 4 min, 35 cycles of 98°C for 10 sec, 60°C for 15 sec and 68°C for 30 sec. Electroporated pCAG-tdTomato was detected by PCR using primers (5’-CGATTACAAGAAGCTGTCCTTCC-3’ and 5’-GCCATGTAGATGGTCTTGAACTC-3’, product size 309 bp), and the following condition: 1 cycle of 98°C for 4 min, 35 cycles of 98°C for 10 sec, 62°C for 15 sec and 68°C for 30 sec. *Actb* was detected by genomic PCR using primers (5’-ATGTACGTAGCCATCCAGGCTGT-3’ and 5’-AGCTGTGGTGGTGAAGCTGTAG-3’, product size 219 bp), and the following condition: 1 cycle of 98°C for 4 min, 35 cycles of 98°C for 10 sec, 60°C for 15 sec and 68°C for 30 sec. Electroporated PSD-95 targeting vector (EGFP), Syp targeting vector (EGFP), and PSD-95 targeting vector (mClover) were detected with primer sets (5’-AATGCAGCCTGCCAGGAAGGAGGTA-3’ and 5’-GACACGCTGAACTTGTGGCCGTTTACG-3’, product size 670 bp, 5’ATGCCCATTCCTGTTCACTCAGCC-3’ and 5’GACACGCTGAACTTGTGGCCGTTTACG-3’, product size 578 bp, 5’-TGCATCCTTGATGTCTCAGCCAATG-3’ and 5’-CGTCCAGCTCGACCAGGATGGGCAC-3’, product size 1320 bp, respectively) and the following condition:1 cycle of 98°C for 4 min, 38 cycles of 98°C for 10 sec, 65°C for 10 sec and 68°C for 30 sec.

RT-PCR

Total RNAs were extracted from mouse brains using RNeasy Mini kit (Qiagen), and reverse-transcribed using SuperScript®III Reverse Transcriptase (Thermo Fisher) according to the manufacturer's instruction. To detect the expression of *PSD-95-mClover* fusion gene, RT-PCR was carried using KOD FX Neo DNA polymerase, primers (5'-ACAACAGCCACCTCTACGGGACC-3' and 5'-CGTCCAGCTCGACCAGGATGGGCAC-3', product size 504 bp), and the following condition: 1 cycle of 98°C for 3 min, 40 cycles of 98°C for 10 sec, 68°C for 40 sec. The expression of *tdTomato* from pCAG-tdTomato was detected by RT-PCR with primers (5'-CGATTACAAGAAGCTGTCCTTCC-3' and 5'-GCCATGTAGATGGTCTTGAACTC-3', product size 309 bp) and the following condition: 1 cycle of 98°C for 3 min, 35 cycles of 98°C for 10 sec, 62°C for 15 sec, 68°C for 30 sec. The expression of *Actb* was detected by RT-PCR with primers (5'-ATGTACGTAGCCATCCAGGCTGT-3' and 5'-AGCTGTGGTGGTGAAGCTGTAG-3', product size 219 bp) and the following condition: 1 cycle of 98°C for 3 min, 30 cycles of 98°C for 10 sec, 60°C for 15 sec, 68°C for 30 sec.

Quantitative RT-PCR was performed on a CFX96 Touch Real-time PCR Detection System (Bio-Rad) using KOD SYBR®qPCR Mix (Toyobo, Osaka, Japan) and the primers described above. All qRT-PCR samples were analyzed in triplicate. The expression level of *PSD-95-mClover* fusion gene for each sample was normalized to the expression of *tdTomato* from pCAG-tdTomato co-electroporated with the CRISPR construct and the targeting vector as a transfection control.

Dark and light adaptation

For preparation of dark-adapted retinas, mice were kept in the dark for 6 hours, and eyes were enucleated under dim red light using Darkroom Safelight (Paterson Photographic Ltd. West Midlands, UK), and fixed in 4% PFA. For preparation of light-adapted retinas, dark-adapted mice were kept under room light (1500 lux) for 30 min, and then eyes were enucleated and fixed.

References

1. Matsuda, T., & Cepko, C.L.
Electroporation and RNA interference in the rodent retina *in vivo* and *in vitro*.

Proc. Natl. Acad. Sci. USA **101**, 16-22 (2004).

2. Matsuda, T., & Cepko, C.L. Controlled expression of transgenes introduced by *in vivo* electroporation. *Proc. Natl. Acad. Sci. USA* **104**, 1027-1032 (2007).
3. Kim, D.S., Matsuda, T., & Cepko, C.L. A core paired-type and POU homeodomain-containing transcription factor program drives retinal bipolar cell gene expression. *J. Neurosci.* **28**, 7748-7764 (2008).
4. Lam, A.J., St-Pierre, F., Gong, Y., Marshall, J.D., Cranfill, P.J., Baird, M.A., McKeown, M.R., Wiedenmann, J., Davidson, M.W., Schnitzer, M.J., Tsien, R.Y., & Lin, M.Z. Improving FRET dynamic range with bright green and red fluorescent proteins. *Nat. Methods* **9**, 1005-1012 (2012).
5. Kanda, T., Sullivan, K.F., & Wahl, G.M. Histone-GFP fusion protein enables sensitive analysis of chromosome dynamics in living mammalian cells. *Curr. Biol.* **8**, 377-385 (1998).
6. Lois, C., Hong, E.J., Pease, S., Brown, E.J., & Baltimore, D. Germline transmission and tissue-specific expression of transgenes delivered by lentiviral vectors. *Science* **295**, 868-872 (2002).
7. Zack, D.J., Bennett, J., Wang, Y., Davenport, C., Klaunberg, B., Gearhart, J., & Nathans, J. Unusual topography of bovine rhodopsin promoter-lacZ fusion gene expression in transgenic mouse retinas. *Neuron* **6**, 187-199 (1991).
8. Ochiai, H., Fujita, K., Suzuki, K., Nishikawa, M., Shibata, T., Sakamoto, N., & Yamamoto, T. Targeted mutagenesis in the sea urchin embryo using zinc-finger nucleases. *Genes Cells* **15**, 875-885 (2010).

Figure S1.

Tagging endogenous rhodopsin with EGFP in the mouse retina.

(**A**) Structures of the mouse *rhodopsin* locus and the knock-in targeting vector to produce a rhodopsin-EGFP fusion protein. The gRNA targeting sequence is underlined and the PAM sequence is shown in red. The predicted Cas9-gRNA cutting position is indicated with a scissor symbol. (**B**) Low magnification views of the retinal section shown in **Fig.1D**. Mouse retinas were co-electroporated at P0 with three plasmids: Rhodopsin targeting vector (EGFP), CAG-mCherry, and CBh-Cas9/U6-gRNA(Rho), and harvested at P21. Retinas were sectioned and stained with anti-GFP (green) and anti-RFP (red) antibodies. Cell nuclei were visualized with DAPI (blue). GFP signal was detected only in rod photoreceptors. ONL, outer nuclear layer; INL, inner nuclear layer; GCL, ganglion cell layer.

Figure S2.

Effect of the length of the homology arms of the donor DNA on knock-in efficiency at the mouse *rhodopsin* locus.

(**A**) Structures of the knock-in targeting vectors to produce a rhodopsin-2A-H2B-EGFP fusion protein, having various lengths of 5' and 3' homology arms. Each targeting vector has the 2kb (pUC)- or 3kb (pBluescript II (pBS))-plasmid backbone. (**B**) Knock-in efficiency of the targeting vectors. Mouse retinas were co-electroporated at P0 with three plasmids: Rhodopsin targeting vector (2A-H2B-EGFP), CAG-H2B-mCherry, and CBh-Cas9/U6-gRNA(Rho). Retinas were harvested at P21, dissociated into single cells, and subjected to FACS analysis to determine the numbers of EGFP- and mCherry-positive cells. Three independently electroporated retinas were analyzed, and relative knock-in efficiency (the number of EGFP/mCherry double-positive cells among the total number of mCherry-positive cells) for each targeting vector was shown. Data represent mean +/- SD (n=3).

Figure S3.

Characterization of the rhodopsin promoter introduced into developing mouse retina by electroporation.

(**A, B**) P0 mouse retinas were electroporated with Rhodopsin promoter-tdTomato and CAG-EGFP. Retinas were harvested at P2, P5, and P8, and sectioned and stained with anti-GFP (green), anti-RFP (red), and anti-phospho-Histon H3 (white, **A**) or anti-Ki67 (white, **B**) antibodies. Cell nuclei were visualized with DAPI (blue). Retinal

cells expressing tdTomato cells were negative for phospho-Histon H3 and Ki67. (**C**) P0 mouse retinas were electroporated with Rhodopsin promoter-Cre, CAG-EGFP, and a Cre-dependent fluorescent reporter, CALNL-tdTomato. Retinas were harvested at P10 when retinogenesis is complete, and sectioned and stained with anti-GFP (green), and anti-RFP (red) antibodies. Cell nuclei were visualized with DAPI (blue). tdTomato-positive signal was detected only in rod photoreceptors in the ONL, indicating that the rhodopsin promoter is not leaky in multipotent retinal progenitors. NBL, neuroblastic layer; ONL, outer nuclear layer; INL, inner nuclear layer; GCL, ganglion cell layer.

Figure S4.

Tagging endogenous glutamine synthetase with mClover in the mouse retina.

(**A**) Structures of the mouse *glutamine synthetase* (*Glul)* locus and the knock-in targeting vector to produce a Glul-EGFP fusion protein. The gRNA targeting sequence is underlined and the PAM sequence is shown in red. The predicted Cas9-gRNA cutting position is indicated with a scissor symbol. (**B**) Isolation of mClover-positive cells by FACS for single-cell genotyping. P0 mouse retinas were electroporated with Glul targeting vector (mClover), CAG-H2B-mCherry, and CBh-Cas9/U6-gRNA (Glul). Retinas were harvested at P21, dissociated into single cells by enzymatic digestion, and mClover/H2B-mCherry double-positive cells were purified by FACS. (**C**) Collected mClover/H2B-mCherry double-positive single cells (Cell G1-G4) in (**B**) were subjected to genomic PCR with the PCR primers shown in (**A**). A mClover-negative single cell (Cell WT) was used as a negative control.

Figure S5.

Tagging endogenous arrestin with EGFP in the mouse retina.

(**A**) Structures of the mouse *arrestin* locus and the knock-in targeting vector to produce an arrestin-EGFP fusion protein. The predicted Cas9-gRNA cutting position is indicated with a scissor symbol. (**B**) Isolation of EGFP-positive cells by FACS for single-cell genotyping. P0 mouse retinas were electroporated with Arrestin targeting vector (EGFP), CAG-H2B-mCherry, and CBh-Cas9/U6-gRNA (arrestin). Retinas were harvested at P21, dissociated into single cells by enzymatic digestion, and EGFP/H2B-mCherry double-positive cells were purified by FACS. (**C**) Collected EGFP/H2B-mCherry double-positive single cells (Cell A1-A4) in (**B**) were subjected to genomic PCR with the PCR primers shown in (**A**). A EGFP-negative single cell (Cell

WT) was used as a negative control. (**D**) Western blot analysis of retinas electroporated with the indicated plasmids at P0 and harvested at P21. Membranes were probed with anti-GFP, anti-arrestin, and anti-RFP antibodies. An arrestin-EGFP fusion protein (~74 kDa) was detected only in the presence of CBh-Cas9/U6-gRNA (arrestin).

Figure S6.

Tagging endogenous synaptophysin with EGFP in the mouse retina.

(**A**) Structures of the mouse *synaptophysin* (*Syp)* locus and the knock-in targeting vector to produce a Syp-EGFP fusion protein. The gRNA targeting sequences are underlined and the PAM sequences are shown in red. The predicted Cas9-gRNA cutting positions are indicated with scissor symbols. These two gRNAs were used together to increase the knock-in efficiency. (**B**) P0 mouse retinas were electroporated with Syp targeting vector (EGFP), CAG-mCherry, and CBh-Cas9 with or without gRNAs for *Syp*, and harvested at P21. DNAs were extracted from the intact retinas, and subjected to PCR analysis. PCR primer sets were used to detect the knock-in allele, the targeting vector, *mCherry*, and endogenous mouse *Actb*, respectively. Lane 1: DNA prepared from the retinas electroporated without CBh-Cas9/U6-gRNA (Syp). Lane2: DNA prepared from the retinas electroporated with CBh-Cas9/U6-gRNA (Syp). Lane3: mouse Syp targeting vector (EGFP) plasmid DNA.

Figure S7.

Tagging endogenous PSD-95 with EGFP in the mouse retina.

(**A**) Structures of the mouse *PSD-95* locus and the knock-in targeting vector to produce a PSD-95-EGFP fusion protein. The gRNA targeting sequences are underlined and the PAM sequences are shown in red. The predicted Cas9-gRNA cutting positions are indicated with scissor symbols. These two gRNAs were used together to increase the knock-in efficiency. (**B**) P0 mouse retinas were electroporated with PSD-95 targeting vector (EGFP), CAG-mCherry, and CBh-Cas9 with or without gRNAs for *PSD-95*, and harvested at P21. DNAs were extracted from the intact retinas, and subjected to PCR analysis. PCR primer sets were used to detect the knock-in allele, the targeting vector, *mCherry*, and endogenous mouse *Actb*, respectively. Lane 1: DNA prepared from the retinas electroporated without CBh-Cas9/U6-gRNA(PSD-95). Lane2: DNA prepared from the retinas electroporated with CBh-Cas9/U6-gRNA(PSD-95). Lane3: mouse PSD-95 targeting vector (EGFP)

plasmid DNA.

Figure S8.

Use of two gRNAs increases knock-in efficiency in the mouse retina

(**A**) Structures of the mouse *synaptophysin* (*Syp)* locus and the knock-in targeting vector to produce a Syp-mClover fusion protein. Two gRNA targeting sequences (gRNA_A and gRNA_B) are underlined and the PAM sequences are shown in red. The predicted Cas9-gRNA cutting positions are indicated with scissor symbols. (**B**) Estimated knock-in efficiency at the mouse *Syp* locus. P0 mouse retinas were electroporated with Syp targeting vector (mClover), CAG-H2B-mCherry, and CBh-Cas9 with single gRNA or two gRNAs for *Syp*, or without gRNA. Retinas were harvested at P18, sectioned and stained with anti-GFP and anti-RFP antibodies. Cell nuclei were visualized with DAPI. Sections were made from two independently electroporated retinas for each DNA sample, and ten sections per retina were analyzed. Knock-in efficiency (the number of mClover-positive cells per the number of mCherry-positive cells) was shown. Data represent mean +/- SD (n=20).

Figure S9.

Tagging endogenous PSD-95 with mClover in the mouse brain.

(**A**) Structures of the mouse *PSD-95* locus and the knock-in targeting vector to produce a PSD-95-mClover fusion protein. The gRNA targeting sequences are underlined and the PAM sequences are shown in red. The predicted Cas9-gRNA cutting positions are indicated with scissor symbols. These two gRNAs were used together to increase the knock-in efficiency. (**B**) RT-PCR analysis of electroporated mouse brains. E14.5 mouse brains were electroporated with mouse PSD-95 targeting vector (mClover), CAG-tdTomato, and CBh-Cas9 with or without gRNAs for *PSD-95*, and harvested at P65. Total RNAs were extracted from the brains, and subjected to RT-PCR analysis. PCR primer sets were used to detect cDNAs encoding *PSD-95-mClover*, *tdTomato*, and endogenous mouse *Actb*, respectively. Lane 1: RNA prepared from the brains electroporated without CBh-Cas9/U6-gRNA (PSD-95). Lanes 2, 3: RNA prepared from the brains electroporated with CBh-Cas9/U6-gRNA (PSD-95). Lane 4: mouse PSD-95 targeting vector (mClover) plasmid DNA. (**C**) Genomic PCR analysis of electroporated mouse brains. E14.5 mouse brains were electroporated with mouse PSD-95 targeting vector (mClover), CAG-tdTomato, and CBh-Cas9 with or without gRNAs for PSD-95, and harvested at P65. DNAs were

extracted from the brains, and subjected to PCR analysis. PCR primer sets were used to detect the knock-in allele, the targeting vector, *tdTomato*, and endogenous mouse *Actb*, respectively. Lane 1: DNA prepared from the brains electroporated without CBh-Cas9/U6-gRNA (PSD-95). Lanes 2, 3: DNA prepared from the brains electroporated with CBh-Cas9/U6-gRNA (PSD-95). Lane 4: mouse PSD-95 targeting vector (mClover) plasmid DNA.

Figure S10.

Full-length images of the agarose gels and immunoblots presented in Figure 6. (**A**) Full-length images of the immunoblots shown in **Fig.6D**. (**B**) Full-length images of the immunoblots shown in **Fig.6G**. (**C**) Full-length images of the agarose gels shown in **Fig.6H**.

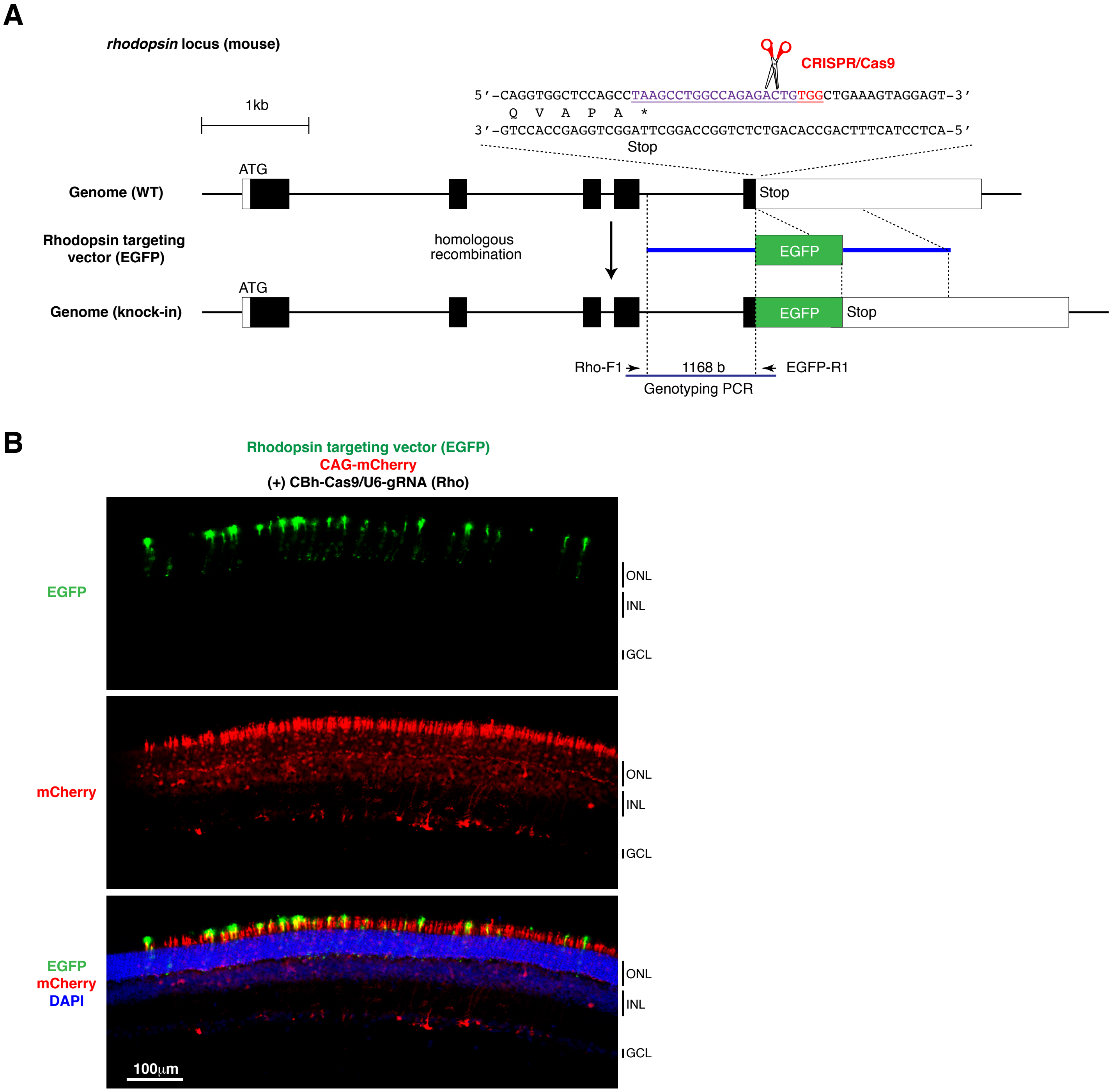
A
rhodopsin locus (mouse)
CRISPR/Cas9
1kb
5'-CAGGTGGCTCCAGCCTAAGCCTGGCCAGAGACTGTGGCTGAAAGTAGGAGT-3'
Q V A P A *
3'-GTCCACCGAGGTCGGATTCGGACCGGTCTCTGACACCGACTTTCATCCTCA-5'
Stop
ATG
Genome (WT)
Stop
Rhodopsin targeting vector (EGFP)
homologous recombination
EGFP
ATG
Genome (knock-in)
EGFP
Stop
Rho-F1
1168 b
EGFP-R1
Genotyping PCR
B
Rhodopsin targeting vector (EGFP)
CAG-mCherry
(+) CBh-Cas9/U6-gRNA (Rho)
EGFP
ONL
INL
GCL
mCherry
ONL
INL
GCL
EGFP
mCherry
DAPI
ONL
INL
GCL
100µm

Fig.S1

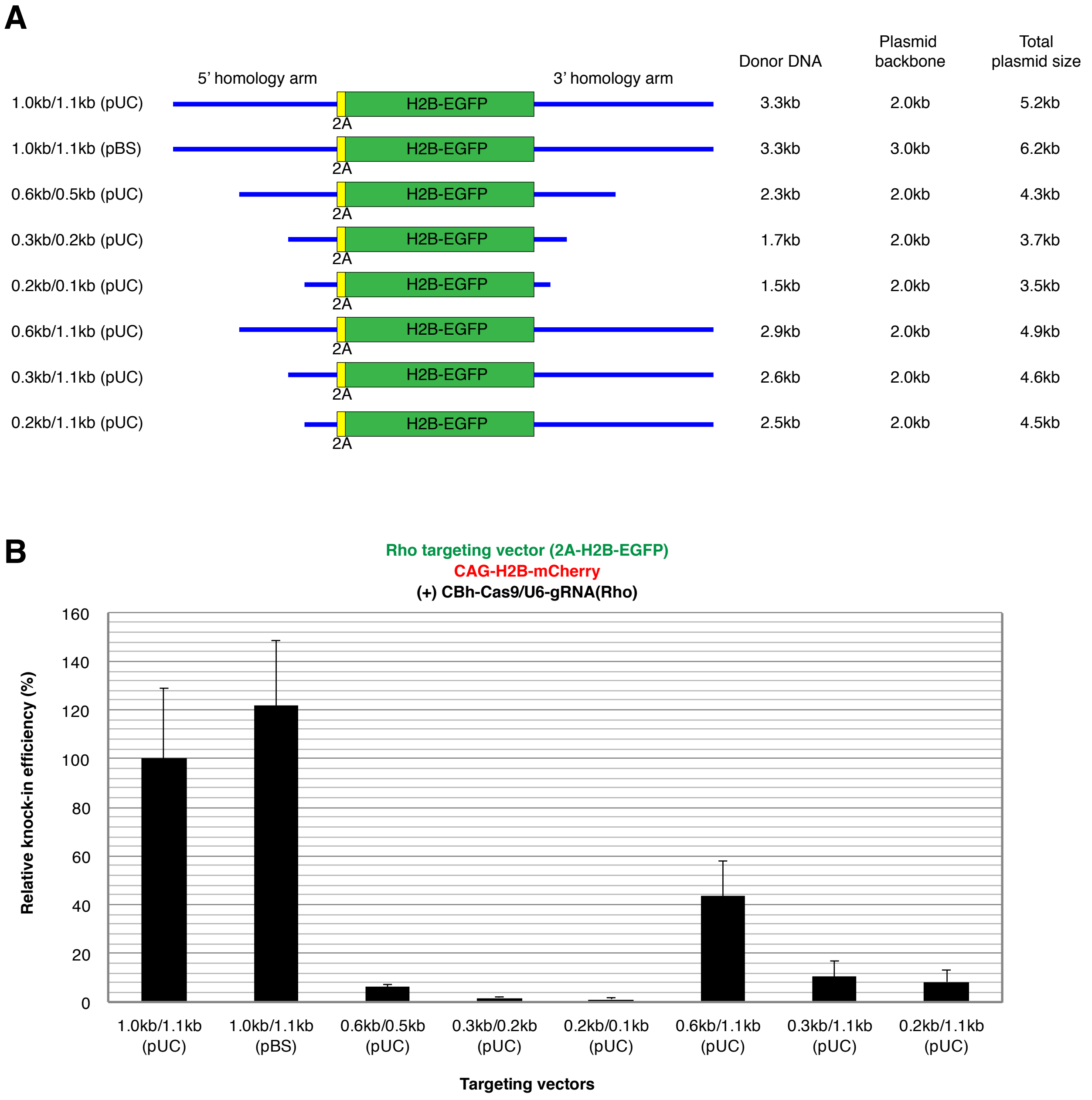
A
5’ homology arm
3’ homology arm
Donor DNA
Plasmid backbone
Total plasmid size
1.0kb/1.1kb (pUC)
H2B-EGFP
2A
3.3kb
2.0kb
5.2kb
1.0kb/1.1kb (pBS)
3.3kb
3.0kb
6.2kb
0.6kb/0.5kb (pUC)
2.3kb
2.0kb
4.3kb
0.3kb/0.2kb (pUC)
1.7kb
2.0kb
3.7kb
0.2kb/0.1kb (pUC)
1.5kb
2.0kb
3.5kb
0.6kb/1.1kb (pUC)
2.9kb
2.0kb
4.9kb
0.3kb/1.1kb (pUC)
2.6kb
2.0kb
4.6kb
0.2kb/1.1kb (pUC)
2.5kb
2.0kb
4.5kb
B
Rho targeting vector (2A-H2B-EGFP)
CAG-H2B-mCherry
(+) CBh-Cas9/U6-gRNA(Rho)
Relative knock-in efficiency (%)
160
140
120
100
80
60
40
20
0
1.0kb/1.1kb (pUC)
1.0kb/1.1kb (pBS)
0.6kb/0.5kb (pUC)
0.3kb/0.2kb (pUC)
0.2kb/0.1kb (pUC)
0.6kb/1.1kb (pUC)
0.3kb/1.1kb (pUC)
0.2kb/1.1kb (pUC)
Targeting vectors

Fig.S2

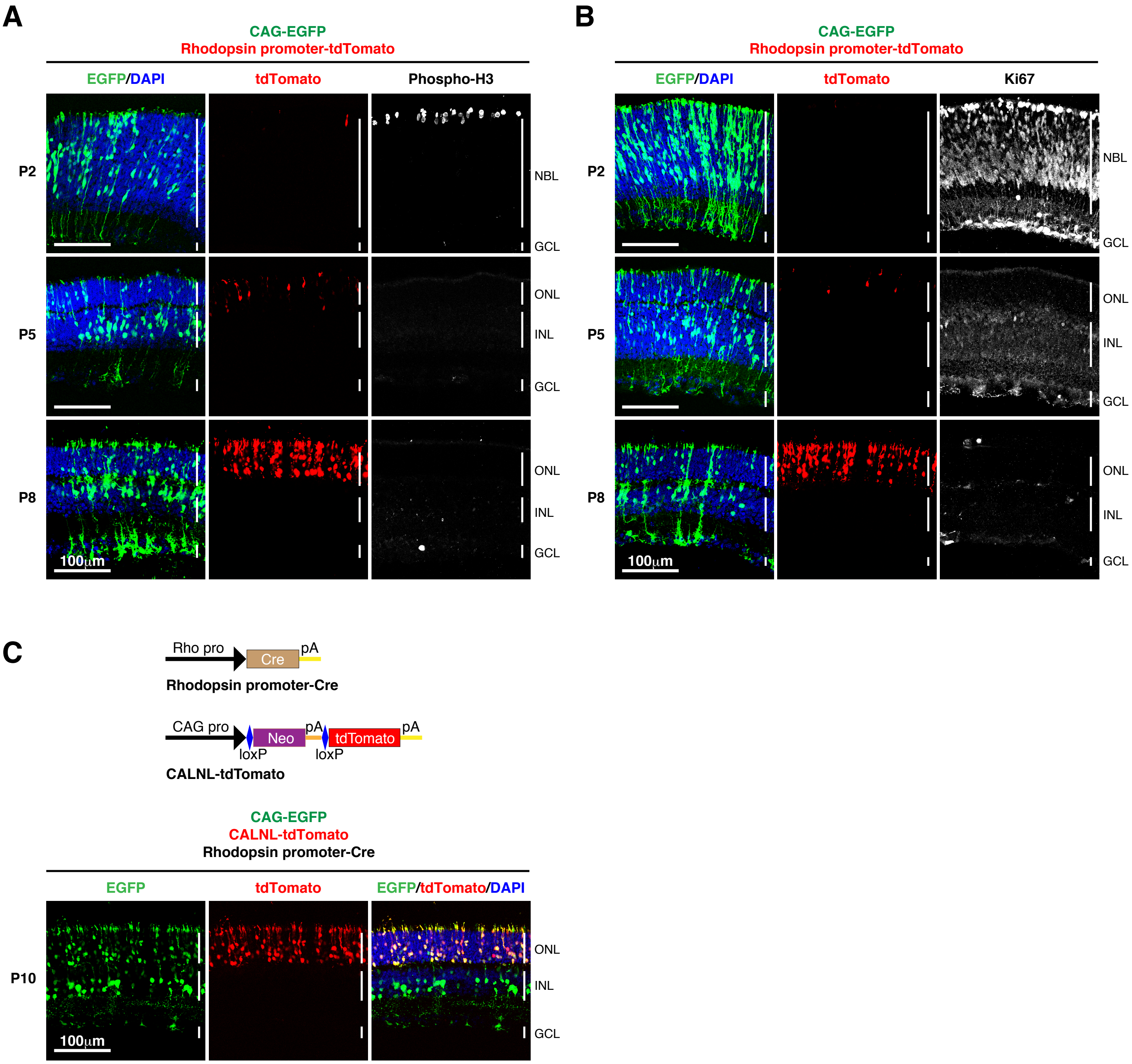
A
CAG-EGFP
Rhodopsin promoter-tdTomato
EGFP/DAPI
tdTomato
Phospho-H3
P2
P5
P8
NBL
GCL
ONL
INL
100μm
B
CAG-EGFP
Rhodopsin promoter-tdTomato
EGFP/DAPI
tdTomato
Ki67
P2
P5
P8
NBL
GCL
ONL
INL
100μm
C
Rho pro
Cre
pA
Rhodopsin promoter-Cre
CAG pro
Neo
pA
tdTomato
pA
loxP
loxP
CALNL-tdTomato
CAG-EGFP
CALNL-tdTomato
Rhodopsin promoter-Cre
EGFP
tdTomato
EGFP/tdTomato/DAPI
P10
ONL
INL
GCL
100μm

Fig.S3

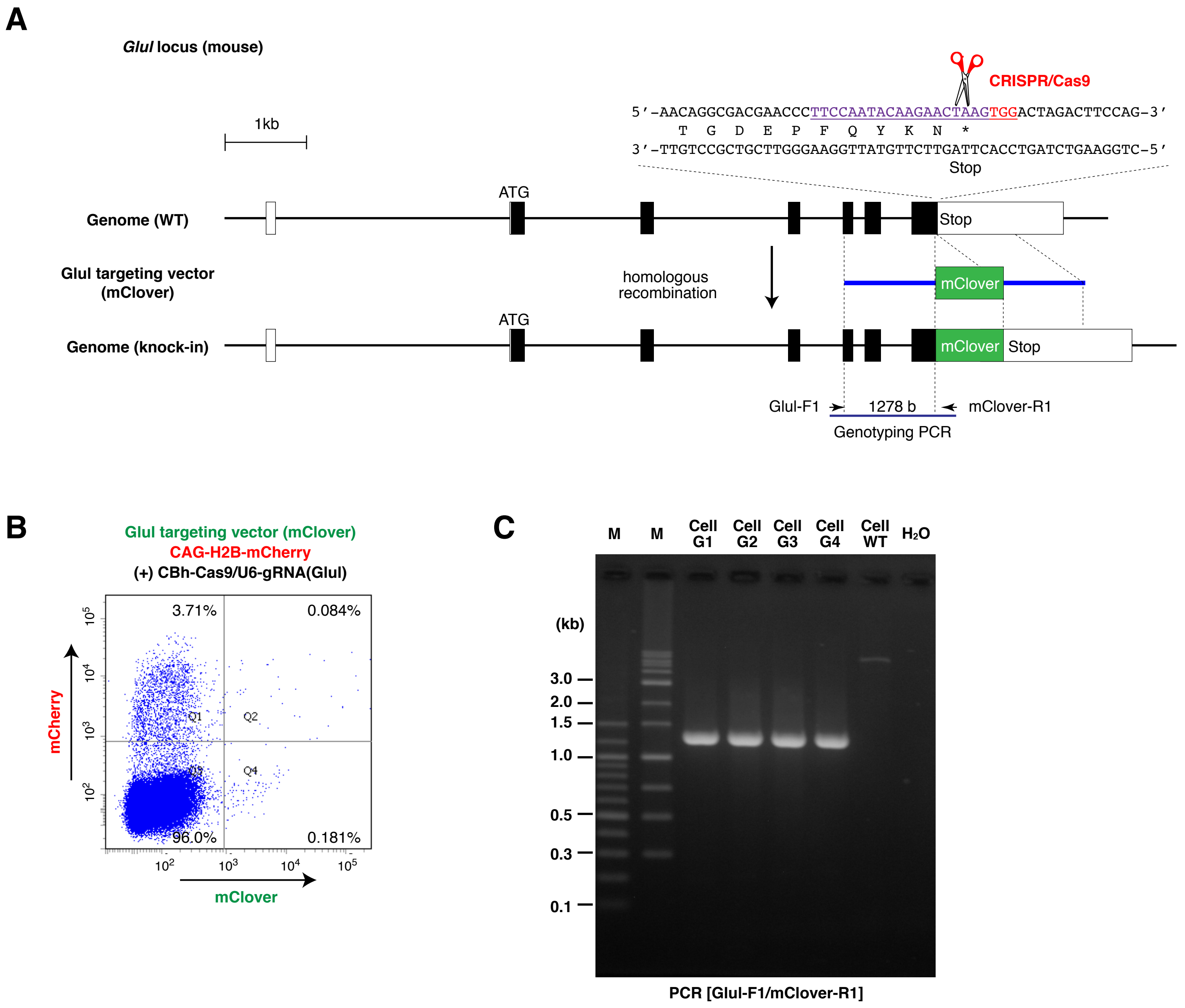
A
Glul locus (mouse)
CRISPR/Cas9
5′-AACAGGCGACGAACCCTTCCAATACAAGAACTAAGTGGACTAGACTTCCAG-3′
T G D E P F Q Y K N *
3′-TTGTCCGCTGCTTGGGAAGGTTATGTTCTTGATTCACCTGATCTGAAGGTC-5′
Stop
1kb
ATG
Genome (WT)
Stop
Glul targeting vector (mClover)
homologous recombination
mClover
ATG
Genome (knock-in)
mClover
Stop
Glul-F1
1278 b
mClover-R1
Genotyping PCR
B
Glul targeting vector (mClover)
CAG-H2B-mCherry
(+) CBh-Cas9/U6-gRNA(Glul)
3.71%
0.084%
96.0%
0.181%
mCherry
mClover
C
M
M
Cell G1
Cell G2
Cell G3
Cell G4
Cell WT
H₂O
(kb)
3.0
2.0
1.5
1.0
0.5
0.3
0.1
PCR [Glul-F1/mClover-R1]

Fig.S4

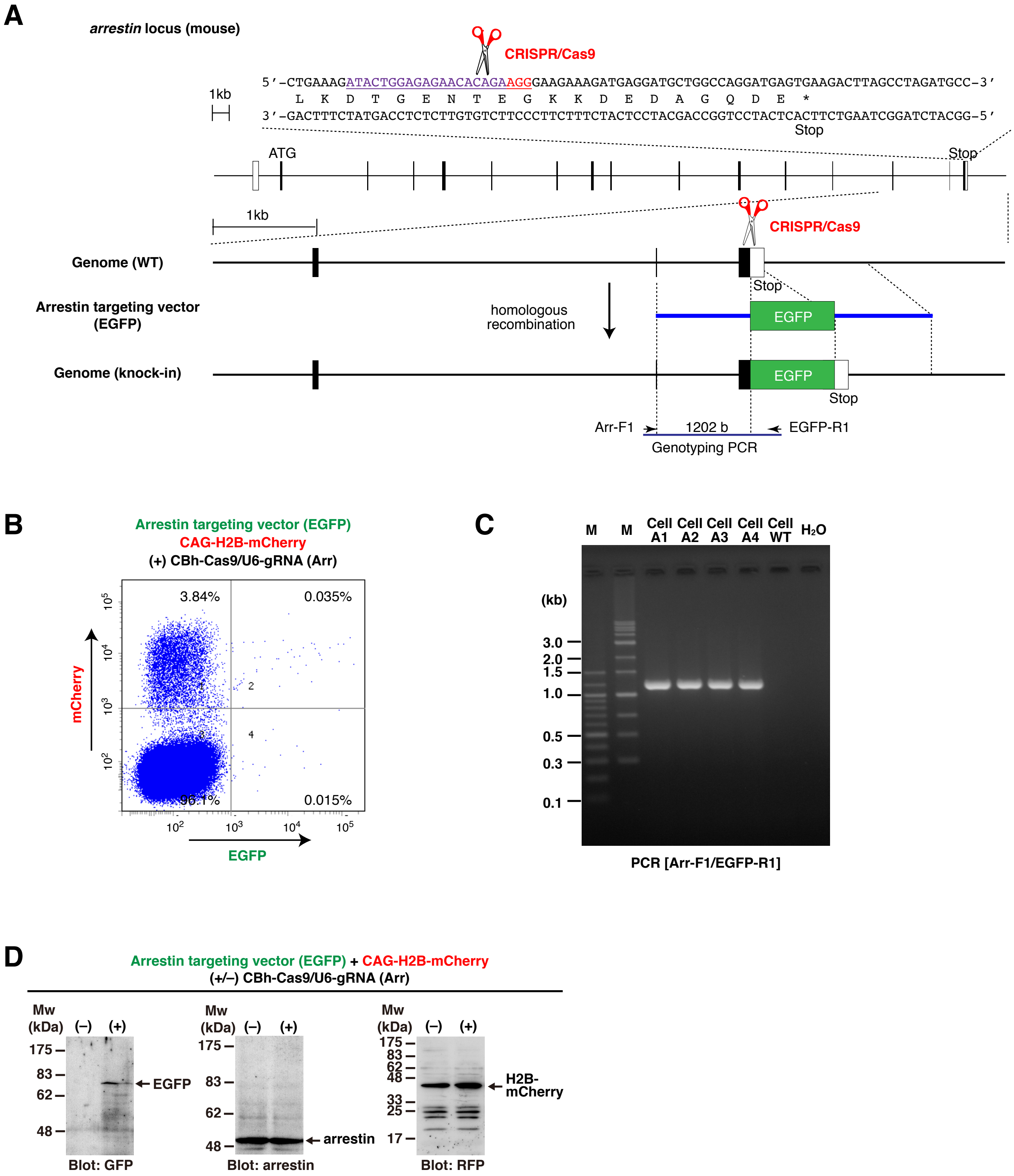
A
arrestin locus (mouse)
CRISPR/Cas9
5'-CTGAAAGATACTGGAGAGAACACAGAAGGGAAGAAAGATGAGGATGCTGGCCAGGATGAGTGAAGACTTAGCCTAGATGCC-3'
L K D T G E N T E G K K D E D A G Q D E *
3'-GACTTTCTATGACCTCTCTTGTGTCTTCCCTTCTTTCTACTCCTACGACCGGTCCTACTCACTTCTGAATCGGATCTACGG-5'
Stop
1kb
ATG
Stop
1kb
CRISPR/Cas9
Genome (WT)
Stop
Arrestin targeting vector (EGFP)
homologous recombination
EGFP
Genome (knock-in)
EGFP
Stop
Arr-F1
1202 b
EGFP-R1
Genotyping PCR
B
Arrestin targeting vector (EGFP)
CAG-H2B-mCherry
(+) CBh-Cas9/U6-gRNA (Arr)
3.84%
0.035%
96.1%
0.015%
mCherry
EGFP
C
M M Cell A1 Cell A2 Cell A3 Cell A4 Cell WT H2O
(kb)
3.0
2.0
1.5
1.0
0.5
0.3
0.1
PCR [Arr-F1/EGFP-R1]
D
Arrestin targeting vector (EGFP) + CAG-H2B-mCherry
(+/–) CBh-Cas9/U6-gRNA (Arr)
Mw (kDa) (–) (+)
175
83
62
48
EGFP
Blot: GFP
Mw (kDa) (–) (+)
175
83
62
48
arrestin
Blot: arrestin
Mw (kDa) (–) (+)
175
83
62
48
33
25
17
H2B-mCherry
Blot: RFP

Fig.S5

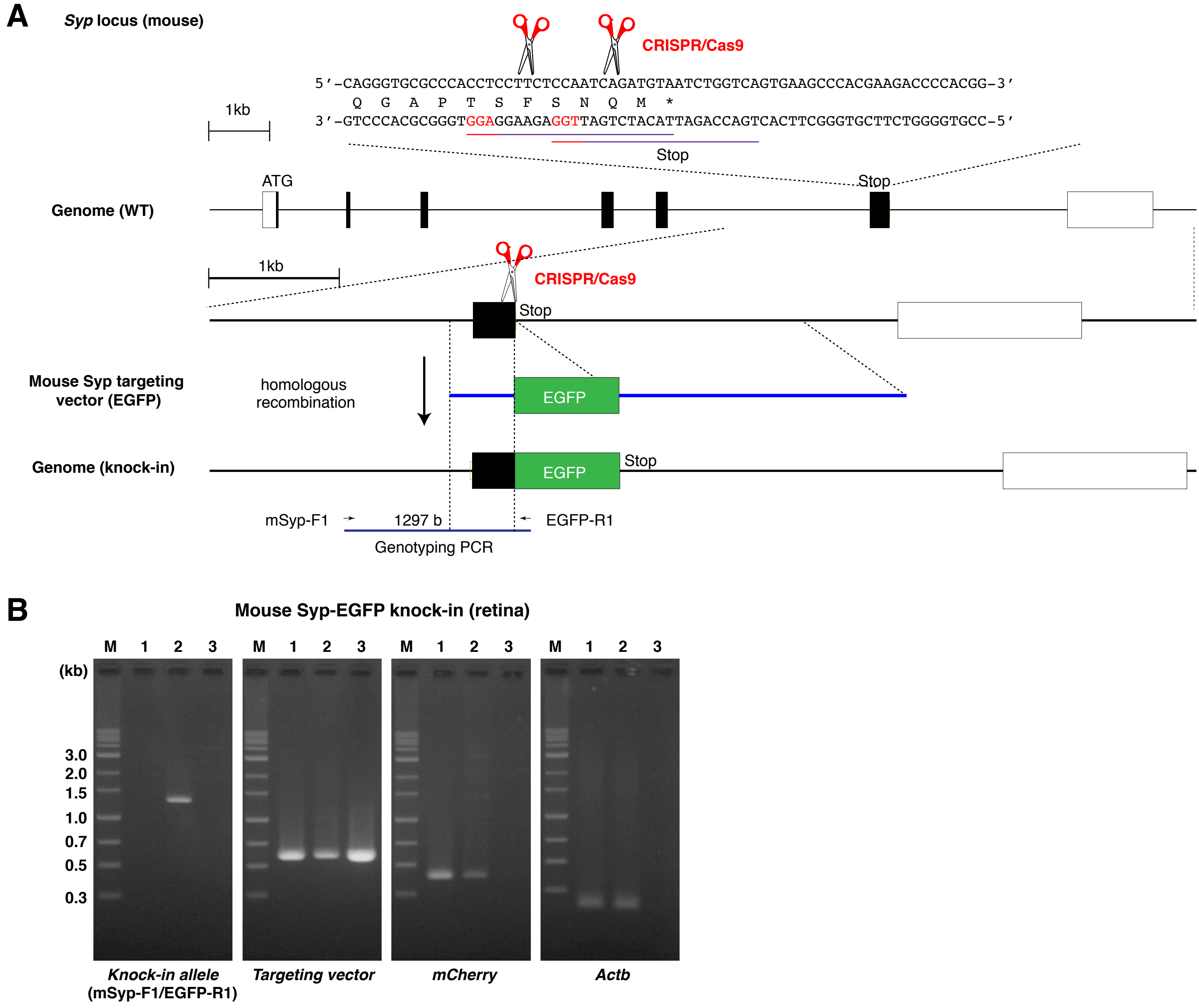
A
Syp locus (mouse)
CRISPR/Cas9
5'-CAGGGTGCGCCCACCTCCTTCTCCAATCAGATGTAATCTGGTCAGTGAAGCCCACGAAGACCCCACGG-3'
Q G A P T S F S N Q M *
3'-GTCCCACGCGGGTGGAGGAAGAGGTTAGTCTACATTAGACCAGTCACTTCGGGTGCTTCTGGGGTGCC-5'
1kb
Stop
ATG
Genome (WT)
1kb
CRISPR/Cas9
Stop
Mouse Syp targeting vector (EGFP)
homologous recombination
EGFP
Genome (knock-in)
EGFP
Stop
mSyp-F1
1297 b
EGFP-R1
Genotyping PCR
B
Mouse Syp-EGFP knock-in (retina)
M 1 2 3 M 1 2 3 M 1 2 3 M 1 2 3
(kb)
3.0
2.0
1.5
1.0
0.7
0.5
0.3
Knock-in allele (mSyp-F1/EGFP-R1)
Targeting vector
mCherry
Actb

Fig.S6

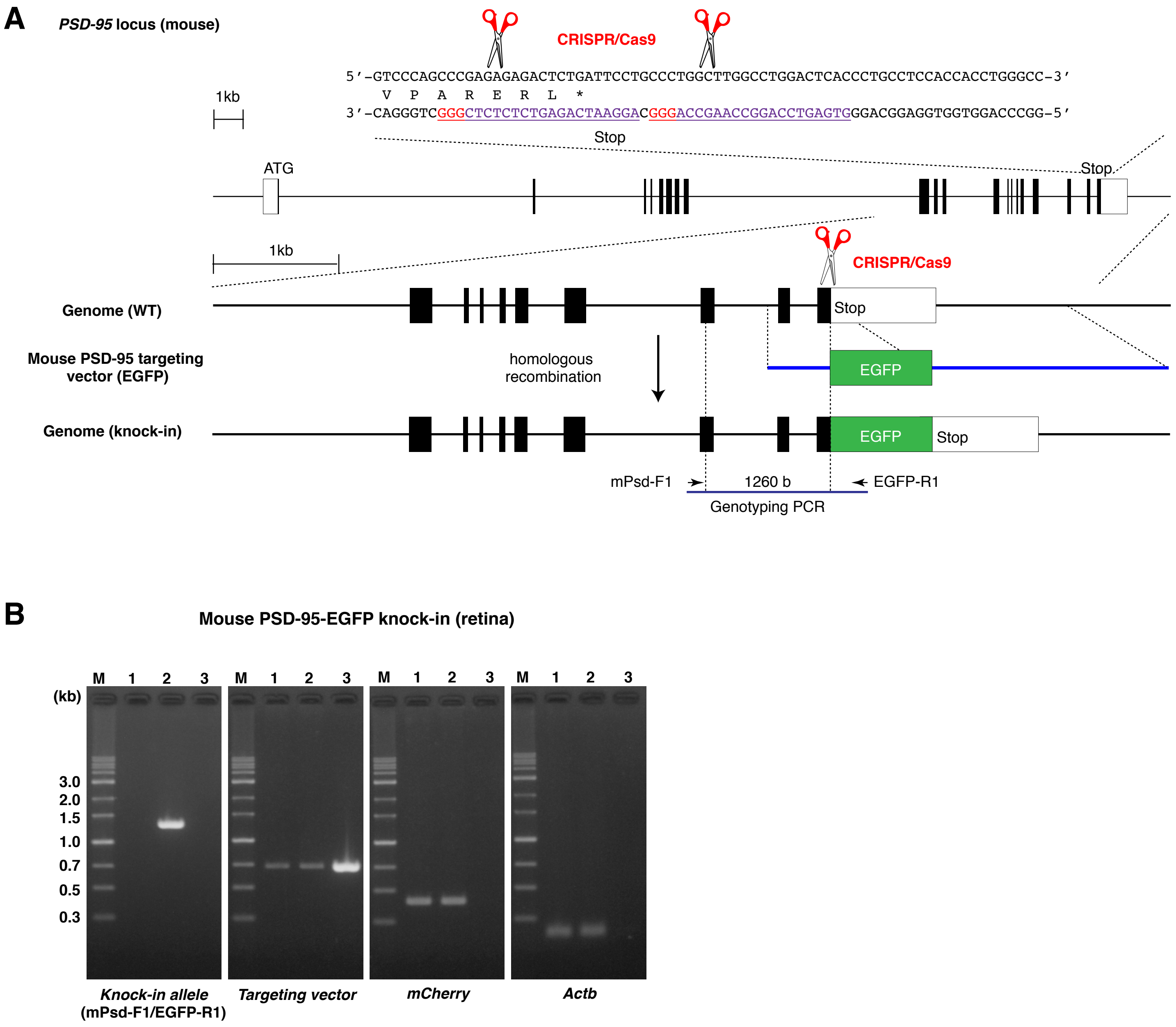
A
PSD-95 locus (mouse)
CRISPR/Cas9
5'-GTCCCAGCCCGAGAGAGACTCTGATTCCTGCCCTGGCTTGGCCTGGACTCACCCTGCCTCCACCACCTGGGCC-3'
V P A R E R L *
3'-CAGGGTCGGGCTCTCTCTGAGACTAAGGACGGGACCGAACCGGACCTGAGTGGGACGGAGGTGGTGGACCCGG-5'
Stop
1kb
ATG
Stop
1kb
CRISPR/Cas9
Genome (WT)
Stop
Mouse PSD-95 targeting vector (EGFP)
homologous recombination
EGFP
Genome (knock-in)
EGFP
Stop
mPsd-F1
1260 b
EGFP-R1
Genotyping PCR
B
Mouse PSD-95-EGFP knock-in (retina)
M 1 2 3
(kb)
3.0
2.0
1.5
1.0
0.7
0.5
0.3
Knock-in allele (mPsd-F1/EGFP-R1)
Targeting vector
mCherry
Actb

Fig.S7

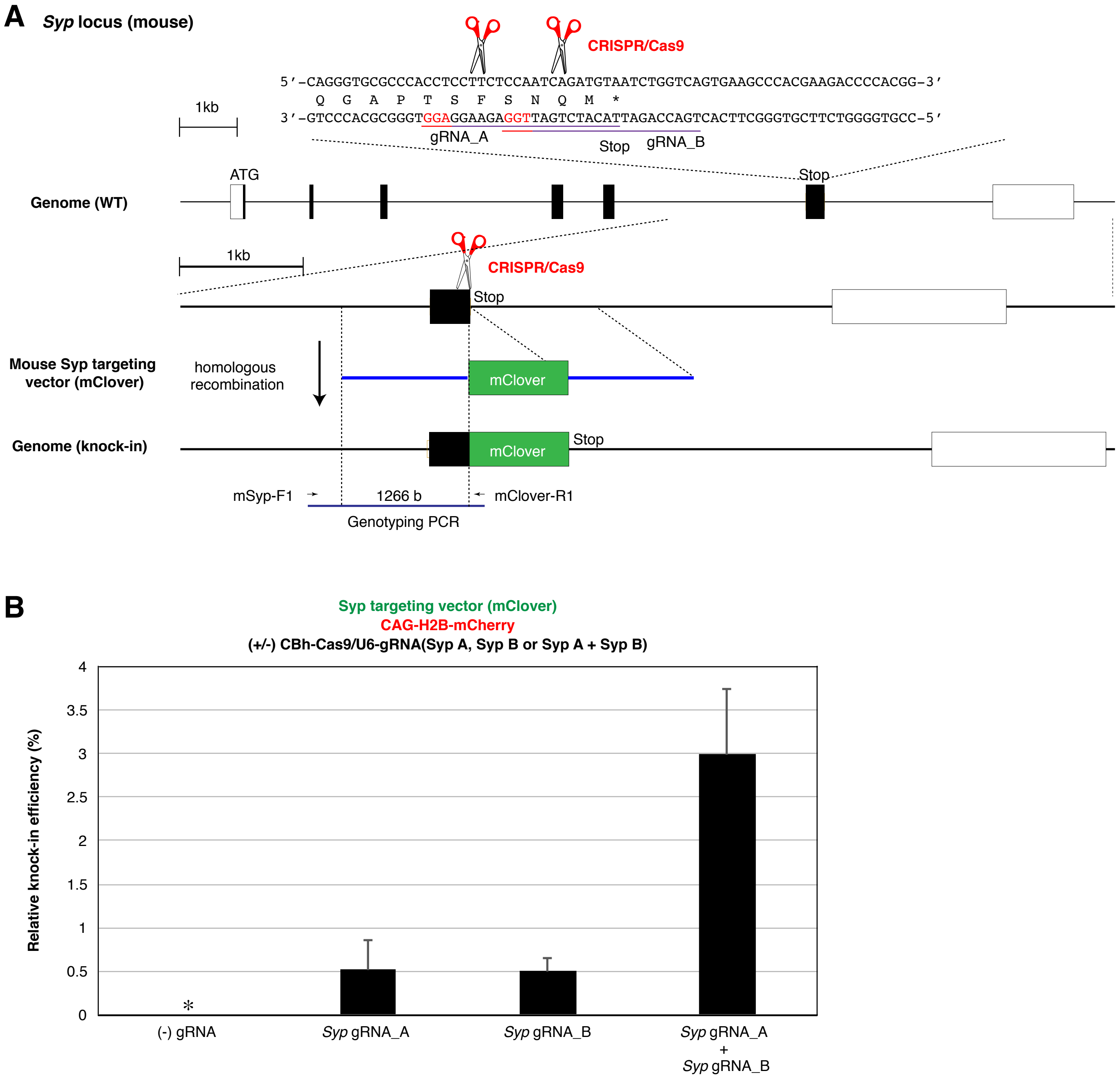
A
Syp locus (mouse)
CRISPR/Cas9
5'-CAGGGTGCGCCCACCTCCTTCTCCAATCAGATGTAATCTGGTCAGTGAAGCCCACGAAGACCCCACGG-3'
Q G A P T S F S N Q M *
3'-GTCCCACGCGGGTGGAGGAAGAGGTTAGTCTACATTAGACCAGTCACTTCGGGTGCTTCTGGGGTGCC-5'
gRNA_A
Stop
gRNA_B
1kb
ATG
Stop
Genome (WT)
1kb
CRISPR/Cas9
Stop
Mouse Syp targeting vector (mClover)
homologous recombination
mClover
Genome (knock-in)
mClover
Stop
mSyp-F1
1266 b
mClover-R1
Genotyping PCR
B
Syp targeting vector (mClover)
CAG-H2B-mCherry
(+/-) CBh-Cas9/U6-gRNA(Syp A, Syp B or Syp A + Syp B)
Relative knock-in efficiency (%)
4
3.5
3
2.5
2
1.5
1
0.5
0
*
(-) gRNA
Syp gRNA_A
Syp gRNA_B
Syp gRNA_A
+
Syp gRNA_B

Fig.S8

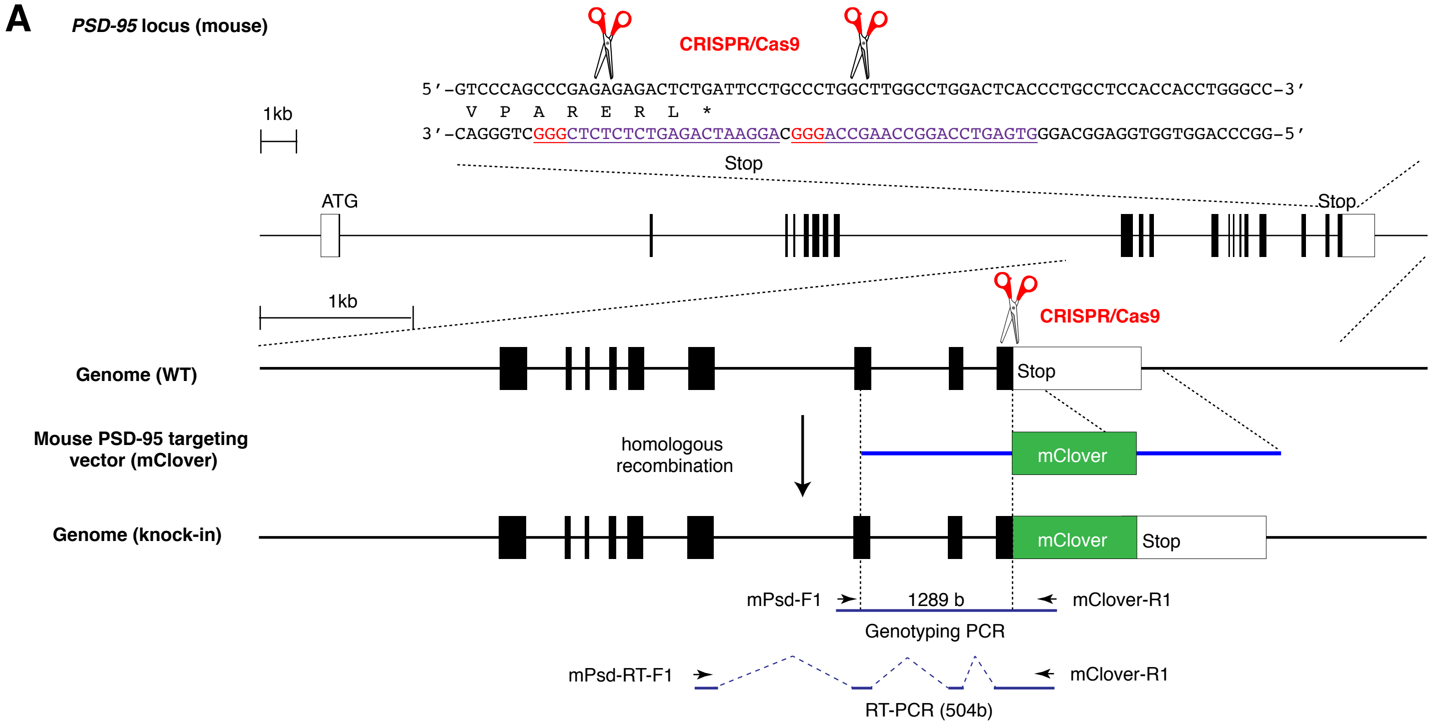
A
PSD-95 locus (mouse)
CRISPR/Cas9
5′-GTCCCAGCCCGAGAGAGACTCTGATTCCTGCCCTGGCTTGGCCTGGACTCACCCTGCCTCCACCACCTGGGCC-3′
V P A R E R L *
3′-CAGGGTCGGGCTCTCTCTGAGACTAAGGACGGGACCGAACCGGACCTGAGTGGGACGGAGGTGGTGGACCCGG-5′
1kb
Stop
ATG
Stop
1kb
CRISPR/Cas9
Genome (WT)
Stop
Mouse PSD-95 targeting vector (mClover)
homologous recombination
mClover
Genome (knock-in)
mClover
Stop
mPsd-F1
1289 b
mClover-R1
Genotyping PCR
mPsd-RT-F1
mClover-R1
RT-PCR (504b)

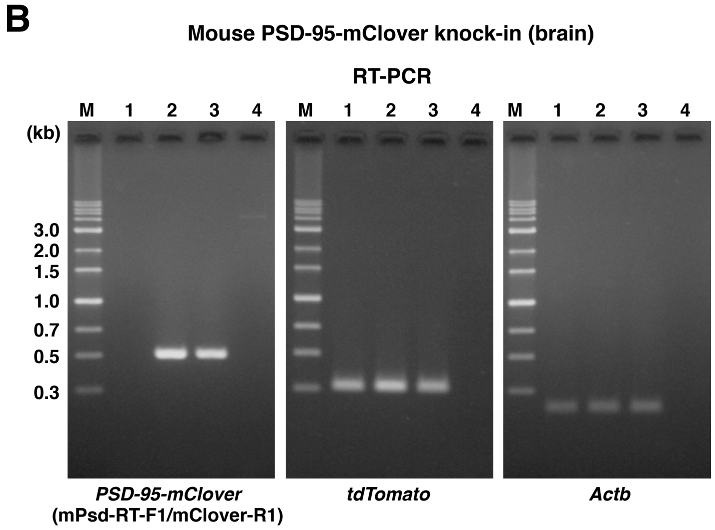
B
Mouse PSD-95-mClover knock-in (brain)
RT-PCR
M 1 2 3 4
M 1 2 3 4
M 1 2 3 4
(kb)
3.0
2.0
1.5
1.0
0.7
0.5
0.3
PSD-95-mClover
(mPsd-RT-F1/mClover-R1)
tdTomato
Actb

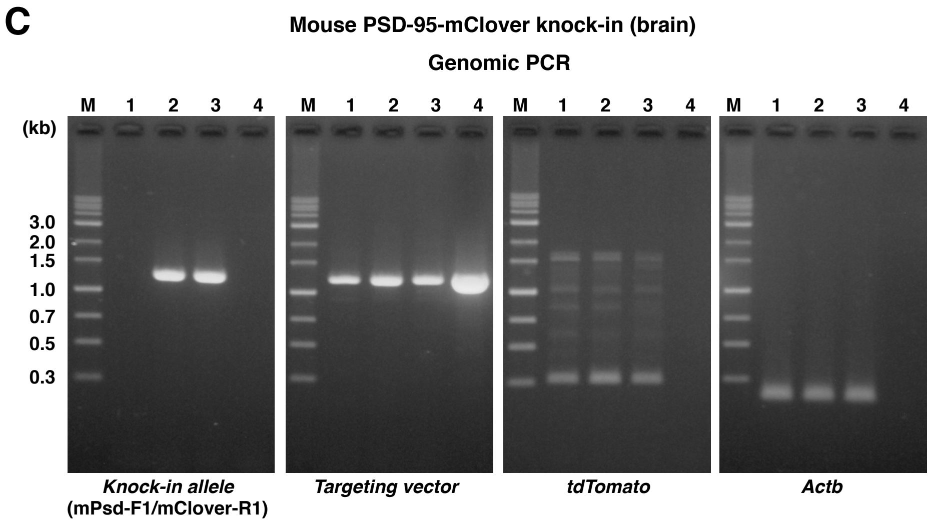
C
Mouse PSD-95-mClover knock-in (brain)
Genomic PCR
M 1 2 3 4
M 1 2 3 4
M 1 2 3 4
M 1 2 3 4
(kb)
3.0
2.0
1.5
1.0
0.7
0.5
0.3
Knock-in allele
(mPsd-F1/mClover-R1)
Targeting vector
tdTomato
Actb

Fig.S9

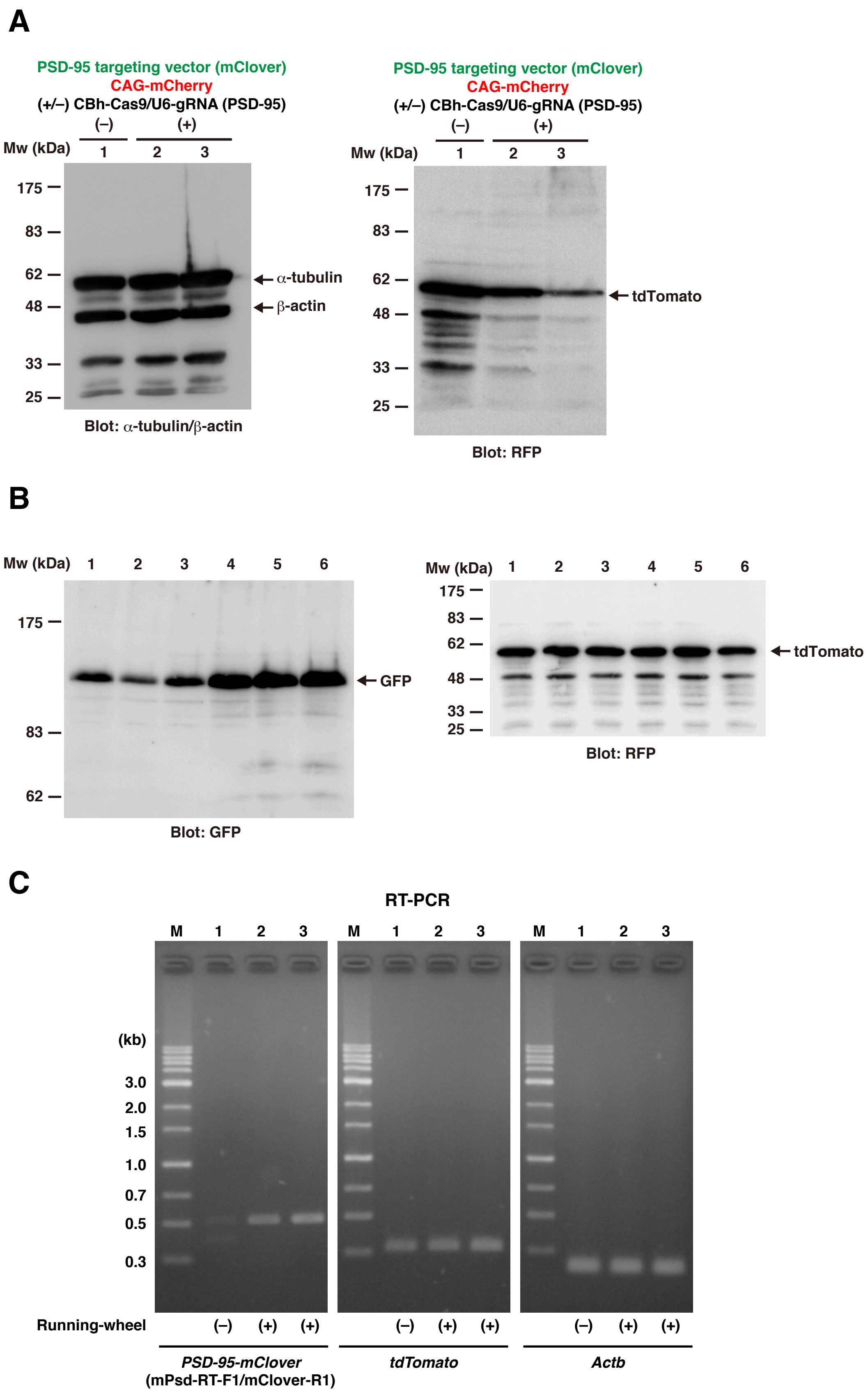
A
PSD-95 targeting vector (mClover)
CAG-mCherry
(+/–) CBh-Cas9/U6-gRNA (PSD-95)
(–)
(+)
Mw (kDa)
1
2
3
175
83
62
48
33
25
α-tubulin
β-actin
Blot: α-tubulin/β-actin
tdTomato
Blot: RFP
B
Mw (kDa)
1
2
3
4
5
6
GFP
Blot: GFP
tdTomato
Blot: RFP
C
RT-PCR
M
(kb)
3.0
2.0
1.5
1.0
0.7
0.5
0.3
Running-wheel
(–)
(+)
(+)
PSD-95-mClover
(mPsd-RT-F1/mClover-R1)
tdTomato
Actb

Fig.S10
